# The N^6^-Methyladenosine-Modified Pseudogene HSPA7 Correlates With the Tumor Microenvironment and Predicts the Response to Immune Checkpoint Therapy in Glioblastoma

**DOI:** 10.3389/fimmu.2021.653711

**Published:** 2021-07-20

**Authors:** Rongrong Zhao, Boyan Li, Shouji Zhang, Zheng He, Ziwen Pan, Qindong Guo, Wei Qiu, Yanhua Qi, Shulin Zhao, Shaobo Wang, Zihang Chen, Ping Zhang, Xing Guo, Hao Xue, Gang Li

**Affiliations:** ^1^ Department of Neurosurgery, Qilu Hospital, Cheeloo College of Medicine and Institute of Brain and Brain-Inspired Science, Shandong University, Jinan, China; ^2^ Shandong Key Laboratory of Brain Function Remodeling, Qilu Hospital of Shandong University, Jinan, China; ^3^ Department of Neurosurgery, Qilu Hospital (Qingdao), Cheeloo College of Medicine, Shandong University, Qingdao, China

**Keywords:** N^6^-methyladenosine, tumor microenvironment, glioblastoma, immune checkpoint blockade, HSPA7

## Abstract

**Background:**

Glioblastoma (GBM), one of the most aggressive tumors of the brain, has no effective or sufficient therapies. Identifying robust biomarkers for the response to immune checkpoint blockade (ICB) therapy, a promising treatment option for GBM patients, is urgently needed.

**Methods:**

We comprehensively evaluated lncRNA m^6^A modification patterns in m^6^A-sequencing (m^6^A-seq) data for GBM tissues and systematically investigated the immune and stromal regulators of these m^6^A-regulated lncRNAs. We used the single-sample gene-set enrichment analysis (ssGSEA) algorithm to investigate the difference in enriched tumor microenvironment (TME) infiltrating cells and the functional annotation of HSPA7 in individual GBM samples. Further, we validated that HSPA7 promoted the recruitment of macrophages into GBM TME *in vitro*, as well as in our GBM tissue section. We also explored its impact on the efficacy of ICB therapy using the patient-derived glioblastoma organoid (GBO) model.

**Results:**

Here, we depicted the first transcriptome-wide m^6^A methylation profile of lncRNAs in GBM, revealing highly distinct lncRNA m^6^A modification patterns compared to those in normal brain tissues. We identified the m^6^A-modified pseudogene HSPA7 as a novel prognostic risk factor in GBM patients, with crucial roles in immunophenotype determination, stromal activation, and carcinogenic pathway activation. We confirmed that HSPA7 promoted macrophage infiltration and SPP1 expression *via* upregulating the YAP1 and LOX expression of glioblastoma stem cells (GSCs) *in vitro* and in our clinical GBM tumor samples. We also confirmed that knockdown of HSPA7 might increase the efficiency of anti-PD1 therapy utilizing the GBO model, highlighting its potential as a novel target for immunotherapy.

**Conclusions:**

Our results indicated that HSPA7 could be a novel immunotherapy target for GBM patients.

## Introduction

Glioblastoma (GBM), one of the most aggressive brain tumors, currently has no effective and sufficient therapies due to its intratumoral heterogeneity and molecular complexity. Immune checkpoint blockade (ICB) therapy is being actively pursued as a promising treatment option for GBM. However, very few patients respond to this therapy (1–5) partly because of the contribution of prominent immunosuppressive factors in the brain tumor microenvironment (TME) in GBM, including tumor-associated macrophages (TAMs), neutrophils, and regulatory T cells (Tregs) (6–8). The development of biomarkers and identification of the definite molecular mechanism underlying resistance to ICB therapy are urgently needed to identify effective therapeutic strategies for GBM.

N^6^-Methyladenosine (m^6^A), the most abundant reversible methylation modification of mRNA, critically affects processes in mRNA metabolism, including splicing, export, translation, and decay. Dysregulation of this modification is clearly linked to diverse pathological processes and disease progression (9, 10), including GBM tumorigenesis (11–15). Recent studies have described the role of m^6^A modification in regulating the immune response (16–19), prompting us to reveal the importance of the spectrum of m^6^A-regulated genes and m^6^A regulatory mechanisms in shaping the TME. Numerous studies have demonstrated that m^6^A is also present in numerous long non-coding RNAs (15, 20–22) (lncRNAs, transcripts longer than 200 nucleotides but lacking functional coding capacity), and lncRNA m^6^A modification has emerged as a fundamental player in cancer progression and immune regulation, suggesting a potential association between the tumor immune response and m^6^A lncRNA modification. However, the lncRNA m^6^A methylation profile has not been systematically clarified in GBM tumors. Additionally, none of these studies have specifically investigated the roles of m^6^A-modified lncRNAs in the overall TME landscape in GBM. Therefore, it is worthwhile to obtain comprehensive knowledge of the cellular TME infiltration characteristics mediated by m^6^A-modified lncRNAs, as this knowledge would contribute to our understanding of the role of m^6^A modification in immune regulation and guide the development of more effective immunotherapeutic strategies.

Here, we depicted the first transcriptome-wide lncRNA m^6^A methylation profile in GBM and normal brain tissues *via* m^6^A sequencing (m^6^A-seq) data analysis, revealing the highly distinct lncRNA m^6^A modification patterns between these two groups. Key immune-stromal-related lncRNAs were identified by differentially expressed gene (DEG) analysis in primary GBM cohorts from The Cancer Genome Atlas (TCGA). Integrating the m^6^A-regulated lncRNAs revealed in this study, we identified HSPA7 as a novel prognostic factor in GBM patients. Through detailed bioinformatic analyses of HSPA7, we identified its crucial role in immunophenotype determination, stromal activation and carcinogenic pathway activation and highlighted its robust capacity to predict the ICB response. We confirmed that HSPA7 facilitated macrophage infiltration *via* the YAP1–LOX axis *in vitro*. We also confirmed that HSPA7 enhanced the efficiency of anti-PD1 therapy utilizing GBM patient-derived glioblastoma organoids, an *ex vivo* model. These results demonstrated that HSPA7 could be a novel immunotherapy target for GBM patients.

## Materials and Methods

### Patient Specimens and Public Patient Cohorts

Human GBM and normal brain tissues for m^6^A-seq were obtained from patients admitted to Qilu Hospital. All participants provided written informed consent, and the research was approved by the Ethics Committee on Scientific Research of Shandong University Qilu Hospital (approval number: KYLL-2018-324).

The RNA sequencing (RNA-seq) transcriptome, somatic mutation data, and corresponding clinicopathological parameters of the TCGA GBM cohort were obtained from the TCGA database (http://cancergenome.nih.gov/). Two Chinese Glioma Genome Atlas (CGGA) GBM RNA-seq datasets and the corresponding clinicopathological parameters were obtained from the CGGA database (http://www.cgga.org.cn/). For ICB data, genomic and clinical information from the IMvigor210 cohort, complete expression data, and detailed clinical annotations were obtained from http://research-pub.Gene.com/imvigor210corebiologies based on the Creative Commons Attribution 3.0 license. For data from GBM patients treated with PD-1 inhibitors (pembrolizumab or nivolumab), clinical information was obtained from Supplementary paper data, and the sequencing data were obtained from SRA PRJNA482620 (2). For patients with melanoma treated with anti-CTLA4 therapy, the expression data were downloaded from the cBioPortal database (http://www.cbioportal.org/), and the detailed clinical characteristics of individual patients were obtained from the supplementary data of a previous paper (23). In addition, the somatic mutation data and information in **Figure S11A** were obtained from the cBioPortal database. The m^6^A-seq sequencing data have been deposited in SRA PRJNA661159 (the data are being processed, submission ID: SUB8069560, released when the paper is published). The processed data are available from the corresponding author upon reasonable request.

### Estimation of TME Cell Characterization

We used the single-sample gene set enrichment analysis (ssGSEA) algorithm to calculate the enrichment score of immune cell infiltration into the GBM TME for each sample. Immune cell-related genes were obtained from Bindea et al. (24) and Robert L (25). (**Table S1**) and included genes related to immune cell types, immune-related pathways and functions. Based on the ssGSEA results, samples from the TCGA GBM cohort were classified into the high immune cell infiltration (immune-H) group or low immune cell infiltration (immune-L) group by using the “hclust” R package.

### Functional Annotation and Pathway Enrichment Analysis

To explore the differences in biological behavior among the samples with distinct HSPA7 expression levels, we used selected HALLMARK (26) and Kyoto Encyclopedia of Genes and Genomes (KEGG) (27) from gene sets from the Molecular Signatures Database (MSigDB) and other commonly used gene signatures (28–30) to estimate pathway enrichment scores for each sample (**Table S2**) by gene set variation analysis (GSVA) using the “GSVA” R package.

The terms enriched with genes positively correlated with the expression of HSPA7 and genes interacting with HSPA7 detected in NCBI (https://www.ncbi.nlm.nih.gov/gene/3311) and the starBase database (http://starbase.sysu.edu.cn/index.php) were analyzed *via* the Metascape resource (http://metascape.org/gp/index.html#/).

### Cell Lines and Reagents

All patient-derived GSC cell lines, including mesenchymal (MES) subtype GSC cell lines (GSC 20 and GSC 267), proneural (PN) subtype GSC cell lines (GSC 8–11), and neural progenitor cells (NPCs) were kindly donated by Dr. Frederick F. Lang and Dr. Krishna P.L. Bhat (The University of Texas, M.D. Anderson Cancer Center, Houston, TX, USA). The cells were cultured in DMEM/F12 supplemented with B27 (Invitrogen,California, USA), 20 ng/ml EGF (R&D Systems, USA), and 20 ng/ml bFGF (R&D Systems, California, USA). The human glioma cell lines U87MG, U251MG, A172, and LN229 and the human monocyte cell line THP-1 were obtained from the Chinese Academy of Sciences Cell Bank. U87MG, U251, A172, and LN229 cells were cultured in DMEM (Thermo Fisher Scientific, USA) supplemented with 10% FBS (Thermo Fisher Scientific; Waltham, MA, USA). THP-1 cells were cultured in RPMI-1640 (Thermo Fisher Scientific) supplemented with 10% FBS (Thermo Fisher Scientific). THP-1 cells were incubated with 100 ng/ml PMA (Sigma-Aldrich; St. Louis, MO, USA) for 24 h *in vitro* to induce their differentiation into macrophages. Cells were cultured in a standard humidified atmosphere of 5% CO_2_ at 37°C.

### Western Blot

Protein was extracted from GSC cells or glioma cells. The following primary antibodies were used: GAPDH (Cell Signaling Technology, Boston, USA, 5174), YAP1 (Cell Signaling Technology, 14074), CD44 (Cell Signaling Technology, 3570), LOX (Abcam, Cambridge, UK, ab174316), and YKL40 (Cell Signaling Technology, 47066).

### Antisense Oligonucleotides, Lentivirus Transfection

ASOs were synthesized by RiboBio (Guangzhou, China). HSPA7/FTO overexpression, METTL3 shRNA and corresponding control lentiviruses were synthesized by GeneChem (Shanghai, China). Target sequences for HSPA7 were as follows: ASO#1: 5′-GGAAGCGGAGCTGAGCAGAT-3′; ASO#2: 5′-CTAACAAGATCACCAATGAC-3′. Target sequences for METTL3 were as follows: 5′-GCCAAGGAACAATCCATTGTT-3′.

### Immunofluorescence

The slides were washed with PBS for 15 min and blocked with 10% goat serum in PBS. The slides were incubated overnight in a humidified chamber at 4°C with the following primary antibodies: LOX (Abcam, ab174316); YAP1 (Cell Signaling Technology, 14074), CD68 (Abcam, ab213363), CD44 (Cell Signaling Technology, 3570), Ki67 (Cell Signaling Technology, 9449), PD-L1 (Abcam, ab213524), and SPP1 (Abcam, ab8448). After primary antibody incubation, the samples were washed with PBS and incubated with the matching fluorescent-conjugated secondary antibody (1:500 dilution, Thermo Fisher) at room temperature for 1 h. Images were captured using a LeicaSP8 confocal microscope (Leica Microsystems, Wetzlar, Germany)

### Fluorescence *In Situ* Hybridization

RNA-FISH was performed according to the instructions of the manufacturer (GenePharma, Shanghai, China). Incubated with a cy3-labeled HSPA7 probe, nuclei were counterstained with DAPI. Images were captured using a LeicaSP8 confocal microscope. Probe sequence: ATCCTTTTGCACCTCCCCGACCC; AACCTTCCCGCACCTTCCCGCCCAGTC.

### Glioblastoma Organoid Model

Glioblastoma organoid models were generated as previously described (31). GBO medium containing 50% DMEM:F12 (Thermo Fisher Scientific), 50% Neurobasal (Thermo Fisher Scientific), 1× GlutaMax (Thermo Fisher Scientific), 1× NEAAs (Thermo Fisher Scientific), 1× PenStrep (Thermo Fisher Scientific), 1× N2 supplement (Thermo Fisher Scientific), 1× B27 w/o vitamin A supplement (Thermo Fisher Scientific), 1× 2-mercaptoethanol (Thermo Fisher Scientific), and 2.5 μg/ml human insulin (Sigma) per well was placed on an orbital shaker rotating at 120 rpm within a 37°C, 5% CO_2_, and 90% humidity sterile incubator.

### Transwell Assay

Transwell assays were performed in 24-well multiwell insert systems according to the protocol of the manufacturer. THP-1 cells were incubated with 100 ng/ml PMA (Sigma-Aldrich) for 24 h *in vitro* to induce their differentiation into macrophages and then added to the top chamber in serum-free media. The bottom chamber was filled with 10% FBS 1640 and GSC growth media. After 24 h of incubation, the top chamber cells were removed using a cotton swab, and the membrane was fixed in 4% paraformaldehyde for 15 min and stained with crystal violet for 15 min. Five fields of adherent cells in each well were photographed randomly.

### Statistical Analysis

Kaplan–Meier survival analysis was performed using GraphPad Prism 7.04, and significant differences between two groups were compared by the log-rank (Mantel–Cox) test. The waterfall function in the “maftools” package was used to visualize the mutational landscape in patients in the high- and low-HSPA7 groups or the high- and low-tumor mutation burden (TMB) groups. Student’s *t*-test was used for two-group comparisons. For comparisons among more than two groups, the Wilcoxon test and one-way ANOVA were used for non-parametric and parametric data (32). P > 0.05 was considered nonsignificance (ns), P ≤ 0.05 was considered statisticallysignificant (*P < 0.05; **P < 0.01; ***P < 0.001, ****P < 0.0001). All data processing with R packages was performed using R Studio (version 3.6.3)

## Results

### Overview of Transcriptome-Wide m^6^A Methylation in lncRNAs in GBM and Normal Brain Tissues

**Figure 1:**
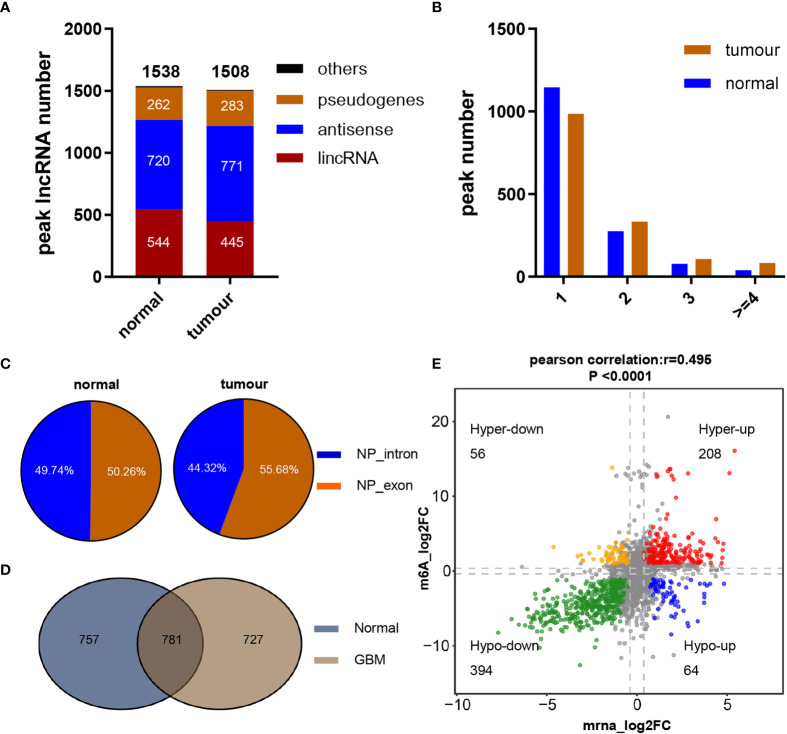
Overview of m^6^A methylation within lncRNAs in GBM and normal brain tissues. **(A)** Numbers and gene types of lncRNAs identified in normal brain and GBM tissue samples as identified by m^6^A-seq. **(B)** The number of m^6^A-modified peaks per lncRNA transcript. **(C)** Distribution of m^6^A peaks across lncRNA transcripts. **(D)** Numbers of common and tissue-specific m^6^A lncRNAs in normal and GBM brain tissues. **(E)** Dot plot of Log2FC (lncRNA expression) *versus* Log2FC (differential m^6^A methylation) values showing a positive correlation between the overall m^6^A methylation level and the lncRNA expression level (Pearson’s r = 0.495; P < 0.0001) and the distribution of genes with significant changes in both the m^6^A (FC ≥ 1.2, P ≤ 0.05) and corresponding lncRNA expression levels in GBM samples compared with normal brain tissues (FC ≥ 2, padj ≤ 0.05).

To understand the pattern of the lncRNA m^6^A modification profiles in GBM, three human GBM tumor tissues and three normal brain tissues were subjected to transcriptome m^6^A-seq, which revealed that a considerable proportion of lncRNAs were extensively m^6^A-modified. In addition, 2,113 m^6^A peaks were identified in the normal group, corresponding to the transcripts of 1,538 genes, including 544 long intergenic ncRNAs (lincRNAs), 720 antisense lncRNAs, 262 pseudogenes, and 12 others. In the GBM group, 2,412 m^6^A peaks were identified, corresponding to the transcripts of 1,508 genes, namely, 445 lincRNAs, 771 antisense lncRNAs, 283 pseudogenes, and 9 other genes (**Figure 1A**). Further analysis showed that most of the lncRNAs (74.5% of the m^6^A-methylated genes in the normal group but 65.3% of the methylated genes in the GBM group) in both groups contained only one peak, while a relatively small number of lncRNAs contained two peaks, and very few lncRNAs contained three or more peaks (**Figure 1B**). Upon further analysis of the distribution profiles of m^6^A peaks within lncRNAs, we found that m^6^A sites were distributed in almost the same proportion in intronic and exonic regions in the normal group but exhibited a slightly increased tendency to be distributed in exonic regions in GBM tissues (**Figure 1C**).

To reveal the significance of m^6^A-methylated lncRNAs in GBM, the differences and overlaps in the m^6^A-modified lncRNAs between the GBM and normal brain groups were analyzed by constructing a Venn diagram. As shown in **Figure 1D**, 781 m^6^A-modified lncRNAs were common to both groups. A total of 727 new genes were expressed, and the expression of 757 genes was lost in the GBM group compared with the normal group, indicating a significant difference in global lncRNA m^6^A modification patterns between the GBM and normal groups. To explore the effect of m^6^A on lncRNA expression, the differentially expressed lncRNAs between 153 primary GBM and five normal brain tissues in the TCGA GBM cohort were compared. Compared with normal samples, GBM tissues exhibited 4,375 differentially expressed lncRNAs (LogFC ≥ 1 and padj ≤ 0.05), with 2,614 upregulated and 1,761 downregulated (**Table S3**). In addition, the global abundance of m^6^A peaks between GBM and normal brain tissues was also compared. Furthermore, integrated analysis of these differentially m^6^A-modified lncRNAs and differentially regulated lncRNAs from the TCGA dataset was conducted. The distribution of genes with a significant change in both the m^6^A level (|FC| ≥ 1.2, P ≤ 0.05) and the overall transcript expression level (|FC| ≥ 2, padj ≤ 0.05) is shown in **Figure 1E**. These genes were divided into four main groups: 208 were hypermethylated and upregulated (“hyperup”), 394 were hypomethylated and downregulated (“hypodown”), 56 were hypermethylated but downregulated (“hyperdown”) and 64 were hypomethylated but upregulated (“hypoup”) in GBM tissues relative to normal brain tissues. We also discovered a positive correlation between differentially methylated m^6^A peaks and the expression levels of their corresponding genes [**Figure 1E**, Pearson correlation coefficient (r) = 0.495, P<0.0001], and the m^6^A modification sites in all of the above four groups of genes were distributed in exonic regions, reconfirming that m^6^A modification can regulate the expression of mature lncRNAs. These results revealed obviously distinct m^6^A modification patterns between GBM and normal brain tissues. Moreover, m^6^A modification could regulate numerous lncRNAs, possibly by regulating their stability, degradation or other functions, as reported previously.

### Identification of the Immune-Stromal-m^6^A-Related Pseudogene HSPA7 as a Novel Prognostic Risk Factor in GBM

**Figure 2:**
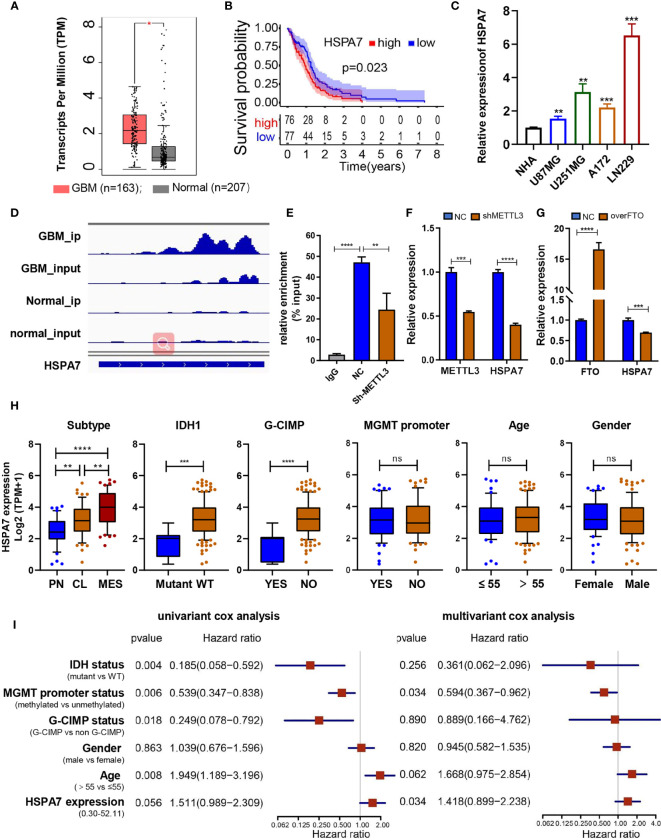
Identification of the immune-stromal-m^6^A-related pseudogene HSPA7 as a novel prognostic factor in GBM. **(A)** The GEPIA database showed that HSAP7 was overexpressed significantly in GBM tissues compared with GETx normal brain tissues. **(B)** Kaplan–Meier survival curves show that HSPA7 is a prognostic risk factor in GBM. **(C)** qPCR assays showing that HSPA7 expression was significantly higher in GBM cells than in NHA cells. Data are presented as the mean ± SD, n = 3. Means were compared with one-way ANOVA, and the NC group is indicated as the control. **(D)** The gene IGV plots of HSPA7 in the m^6^A-seq. **(E)** MeRIP assay showing that HSPA7 was highly enriched by the m^6^A antibody, and the modification can be regulated by the m^6^A methyltransferase METTL3. Data represent mean ± S.D. from three independent experiments. qPCR assay showing that the expression of HSPA7 can be regulated by **(F)** m^6^A methyltransferase METTL3 and **(G)** demethylase FTO. Data represent mean ± S.D. from three independent experiments. **(H)** Distribution of HSPA7 expression in different cohorts stratified by molecular subtype (CL, n = 56; MES, n = 51; PN, n = 44; PN *vs* MES, P < 0.0001; CL *vs* MES, P = 0.0020; PN *vs* CL, P = 0.0013), IDH1 status (mutant, n = 8; WT, n = 141; P = 0.0002), G-CIMP status (G-CIMP, n = 9; non-G-CIMP, n = 142; P < 0.0001), MGMT promoter status (methylated, n = 55; unmethylated, n = 67; P = 0.7445), age (high, age > 55, n = 100; low, age ≤ 55, n = 53; P = 0.5114), and sex (female, n = 54; male, n = 99; P = 0.3996). **(I)** Univariate and multivariate Cox regression analyses of HSPA7 and other clinical features in the overall survival of GBM samples. The statistical significance is shown as: ns, P > 0.05; *P < 0.05; **P < 0.01; ***P < 0.001; ****P < 0.0001.

To investigate the effects of these m^6^A-regulated lncRNAs on cell infiltration into the TME, we assessed the tumor purity, stromal score, and immune score of 153 primary GBM cases in the TCGA GBM cohort using the ESTIMATE algorithm (see *Materials and Methods*, **Table S4**). Then, we performed differential analysis of all RNA-seq data from these 153 GBM samples in the TCGA database based on the median cutoff immune/stromal scores. The volcano plot of the high/low stromal/immune scores revealed differential gene expression profiles between the samples. A total of 791 upregulated lncRNAs and 459 downregulated lncRNAs (|FC| ≥ 1.5, padj ≤ 0.05) were identified based on the difference in immune scores (**Supplementary Figure S1A** and **Table S5**). Simultaneously, 734 upregulated lncRNAs and 269 downregulated lncRNAs (|FC| ≥ 1.5, padj ≤ 0.05) were identified based on the differential analysis of stromal scores (**Figure S1B** and **Table S6**). As the Venn diagram (**Supplementary Figures S1C, D**) indicates, four identical upregulated genes and 11 identical downregulated genes were related to immune activation, stromal activation, and m^6^A modification. Then, we performed Kaplan–Meier analysis on patients stratified by the expression levels of these 15 differentially expressed genes, and only HSPA7 (**Figure 2B**) and AC011899.9 (**Supplementary Figure S2B**) were found to have prognostic significance (P ≤ 0.05) in the 153 TCGA GBM samples based on the median expression levels. The Gene Expression Profiling Interactive Analysis (GEPIA) database was used to show that HSPA7 was significantly overexpressed in GBM tissues compared with normal brain tissues in the Genotype-Tissue Expression (GTEx) database (**Figure 2A**), while AC011899.9 was not (**Supplementary Figure S2A**). Thus, we focused only on HSPA7 in the following work.

We then tested the RNA expression level of HSPA7 in GBM cell lines (U87MG, U251MG, A172, and LN229 cells), and discovered that its expression was significantly higher in GBM cell lines than in normal human astrocyte (NHA) cells (**Figure 2C**). The m^6^A methylation peak distribution and abundance in HSPA7 transcripts from GBM and normal brain tissues, as detected by m^6^A-seq, were visualized using IGV software (**Figure 2D**). We found that HSPA7 was highly enriched in the m^6^A-precipitated fraction, and the m^6^A modification enrichment level could be regulated by methyltransferase-like 3 (METTL3), which contains a catalytic activity domain to catalyze m^6^A formation (**Figure 2E**). Additionally, the expression of HSPA7 was significantly inhibited by knocking down METTL3 (**Figure 2F**) and overexpressing FTO, an m^6^A demethylase (**Figure 2G**). These results provided evidence that HSPA7 harbored high m^6^A modification levels and thus could be regulated in an m^6^A-dependent manner.

To explore the association between HSPA7 expression and clinical characteristics, we first compared HSPA7 expression levels in patients in the TCGA GBM cohort stratified separately by molecular subtype, IDH1 status, CpG island methylator phenotype (G-CIMP) status, MGMT promoter status, age, and sex. As shown in **Figure 2H**, HSPA7 expression in the PN subtype was significantly lower than the HSPA7 expression in the classical (CL) and MES subtypes and was highest in mesenchymal samples. HSPA7 expression was lower in samples with IDH mutations than in samples with wild-type IDH1. Regarding the G-CIMP status, HSPA7 expression was lower in patients with G-CIMP tumors than in those without G-CIMP tumors. No obvious correlations between HSPA7 expression and MGMT promoter methylation, age, or sex were observed. Furthermore, univariate Cox regression analysis of the overall survival of GBM patients in the TCGA cohort showed that high HSPA7 expression (HR: 1.511, P = 0.056) was an independent risk factor associated with the prognosis of GBM. Moreover, high HSPA7 expression (HR: 1.418, P = 0.034) remained a statistically significant factor in GBM patients after adjustment for age, sex, IDH status, MGMT promoter methylation status, and G-CIMP status in subsequent multivariate Cox regression analysis (**Figure 2I**). These results indicated that the pseudogene HSPA7 is a novel risk prognostic biomarker and indicates therapeutic outcomes of GBM patients.

### HSPA7 Is Correlated With Immunophenotypes and TME Landscapes

**Figure 3:**
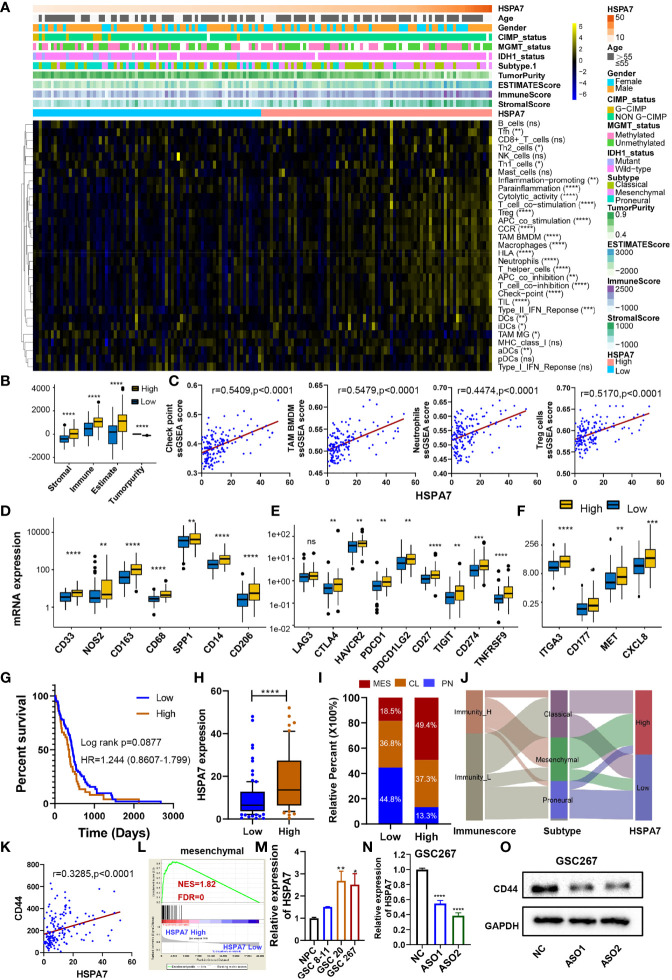
HSPA7 is correlated with immunophenotypes and TME landscapes. **(A)** The enrichment scores of immune cell types and immune-related function-related gene sets were calculated *via the* ssGSEA algorithm. A heatmap was used to visualize these immune characteristics between the HSAP7 high and low expression groups; yellow represents a high enrichment level, black represents a median enrichment level, and blue represents a low enrichment level. **(B)** High expression of HSPA7 presented significantly increased stromal, immune, and ESTIMATE scores and decreased tumorigenicity. **(C)** HSPA7 positively correlated with immunosuppression regulators (checkpoints, macrophages, neutrophils, and Tregs). High expression of HSPA7 presented significantly increased markers of **(D)** suppressor function of myeloid lineages, **(E)** immune inhibitory checkpoints and **(F)** major neutrophil-recruiting chemokines and their receptors. The asterisks indicate a significant statistical p-value calculated using the non-parametric Wilcoxon test (*P < 0.05; **P < 0.01; ***P < 0.001, ****P < 0.0001). **(G)** Compared to the immune-L group (102 patients), patients in the immune-H group (51 patients) experienced poorer prognoses. **(H)** HSPA7 expression was higher in the immune-H group than in the immune-L group. **(I)** The proportion of GBM molecular subtypes in the low and high HSPA7 groups. MES subtype, red; CL subtype, orange; PN subtype, blue. **(J)** Alluvial diagram showing the changes in immune phenotypes, GBM molecular subtypes, and HSPA7 expression. **(K)** HSPA7 positively correlated with CD44, a marker of the MES subtype. **(L)** GSEA of mesenchymal signatures showed that GBM samples with high HSPA7 expression were enriched in the MES subtype compared to GBM samples with low HSPA7 expression. NES, normalized enrichment score; FDR, false discovery rate. **(M)** qPCR assays showed that HSPA7 expression was higher in MES subtype GSCs (GSCs 20 and 267) than in PN subtype GSCs (GSCs 8–11) and neural stem cells (NPCs). **(N)** qPCR assays verified the knockdown efficiency of HSPA7. **(O)** Western blot assays showed that HSPA7 promoted the expression of CD44. The statistical significance is shown as: ns, P > 0.05; *P < 0.05; **P < 0.01; ***P < 0.001; ****P < 0.0001.

To gain further insight into the exact role of HSPA7 in immunophenotype determination, we analyzed 31 immune-associated gene sets representing diverse immune cell types, functions, and pathways (see *Materials and Methods*). As shown in **Figures 3A, B**, the HSPA7-high expression group had significantly greater cell infiltration into the TME, higher immune and stromal scores, and lower tumor purity than the HSPA7-low expression group, confirming that HSPA7 could indeed regulate immune cell infiltration and immune-related gene expression. We then explored the specific differences in 31 immune cell phenotypes with high and low HSPA7 expression. Compared to tumors with low HSPA7 expression, tumors with high HSPA7 expression exhibited significantly increased infiltration of immunosuppressive cell populations, such as macrophages, neutrophils, and Tregs. However, some immune-activating cells, including activated dendritic cells (aDCs), immature DCs (iDCs), plasmacytoid DCs (pDCs), and tumor-infiltrating lymphocytes (TILs), were also enriched (**Supplementary Figure S3A**), indicating the complexity of the TME, in which GBM cells elicit multiple biological behavioral changes through direct or indirect interactions with other TME components. Two recent publications (7, 8) on single-cell mapping of human brain cancer reported that tumor-associated macrophages (TAMs) are the most abundant cellular components in brain TMEs, which can be subdivided ontogenetically into tissue-resident microglia (MGs) and macrophages of embryonic origin, and bone marrow-derived macrophages (BMDMs), showing tumor-promoting and immunosuppressive functions. To identify specific macrophage cell populations linked to HSPA7 expression in GBM, we examined the TCGA GBM dataset for TAM MG and TAM BMDM using validated gene set signatures (25), which demonstrated that tumors with high HSPA7 expression exhibited significantly increased infiltration of TAM BMDMs, while TAM MG was significantly decreased (**Figure 3A** and **Supplementary Figure S3A**), suggesting that HSPA7 enhanced recruitment of tumor-promoting BMDMs into the GBM TME rather than MG. These studies also showed that both activation and exhaustion of lymphocytes were prevalent, with increased relative frequencies of Tregs in the brain tumor TME. Moreover, we found elevated neutrophil infiltration in tumor tissues, revealing the complexity and multifaceted functions of the brain TME. We found that HSPA7 had a significant positive correlation with the enrichment scores of the three immunosuppressive immune cells and immune checkpoints (**Figure 3C**). We next investigated chemokines and immune modulators associated with immune suppression states. We then explored the specific differences in markers of myeloid lineages with suppressive functions (CD33, NOS2, CD163, CD68, SPP1, CD14, CD206), immune inhibitory checkpoints (LAG3, CTLA4, HAVCR2, PDCD1, PDCD1LG2, CD27, TIGIT, CD274, and TNFRSF9), and major neutrophil-recruiting chemokines and their receptors (ITGA3, CD177, MET, and CXCL8) between patients with high and low HSPA7 expression. Tumors with high HSPA7 expression exhibited significantly increased expression levels of these markers compared to tumors with low HSPA7 expression (**Figures 3D–F**), and positive correlations were found between HSPA7 expression and these molecules (**Supplementary Figures S4A–C**). Furthermore, to explore the direct involvement of the HSPA7 in the biological pathway causing immune suppression, we then analyzed myeloid cell-derived macrophage-restricted chemokines, which were the main factors that cause immunosuppression in GBM. We found that compared to tumors with low HSPA7 expression, tumors with high HSPA7 expression exhibited significantly increased myeloid cell-derived macrophage-restricted chemokines (8, 25) (**Supplementary Figures S4D, E**), including CCL17, CXCL2, CXCL3, and CXCL16 (involved in wound healing); immunosuppressive cytokines IL-10, CTSB, and CTSW (participating in multiple tumor-promoting processes, including invasion and metastasis); CCL2 and CCR2 (involved in macrophage chemotaxis); and other chemokine contributions of myeloid cell populations to the inflammatory TME milieu, indicating that HSPA7 could promote immunosuppressive phenotypes and suppress intratumoral antitumor immune responses.

To further understand the exact role of HSPA7 in determining the TME profile and immunophenotype, we performed unsupervised consensus clustering based on the TME cell populations and immune-related functional gene sets identified by Bindea et al. (24). This analysis identified a TME pattern with two clusters corresponding to a TME immune-low (immune-L) and a TME immune-high (immune-H) phenotype (**Supplementary Figure S3B**). Patients in the immune-H group (n = 51) exhibited poorer prognosis (**Figure 3G**) and higher HSPA7 expression levels (**Figure 3H**) than patients in the immune-L group (n = 102). The PN, CL, and MES subtypes of GBM have been most consistently described in the literature; the PN subtype is related to a more favorable outcome, and the MES subtype is related to poorer survival (23). The association between the MES gene expression signature, characterized by NF1 mutation, with reduced tumor purity, elevated invasion, enhanced migration capacity, and infiltration of immunosuppressive cells (macrophages, microglia, mesenchymal stem cells, or other cells) has been identified as a common theme across cancers (33, 34). Our results showed that most MES GBM samples had high expression of HSPA7 as well as the immune-H phenotype (**Figures 3I, J**). Moreover, we observed the same results as all GBM samples by analyzing MES glioblastoma alone, in which tumors with high HSPA7 expression exhibited significantly increased infiltration of immunosuppressive cell populations and regulators, such as macrophages, TAM BMDMs, neutrophils, Tregs, and immune checkpoints (**Supplementary Figures S5A, B**). As MES glioblastomas are strongly associated with higher immunosuppressive cell infiltration, we hypothesized that HSPA7 activated the immune microenvironment by promoting the phenotypic transformation of the GBM MES subtype. As shown in **Supplementary Figure S5C**, compared to tumors with low HSPA7 expression, tumors with high HSPA7 expression exhibited significantly increased expression levels of genes in the MES phenotype signature genes (33). Furthermore, HSPA7 positively correlated with CD44, a marker of the MES subtype (**Figure 3K**). GSEA also showed that GBM samples with high HSPA7 expression were enriched in the MES subtype compared to GBM samples with low HSPA7 expression (**Figure 3L**). In addition, we found that HSPA7 expression was higher in MES GSCs than in PN GSCs (**Figure 3M**). Moreover, Western blot assays revealed that knockdown of HSPA7 reduced the expression of CD44 (**Figures 3N, O**). Overall, these results indicated that in GBM, HSPA7 may be a robust indicator of the immunophenotype and be significantly correlated with a poorer immune response.

### HSPA7 Is Correlated With Stromal and Carcinogenic Activation Pathways

**Figure 4:**
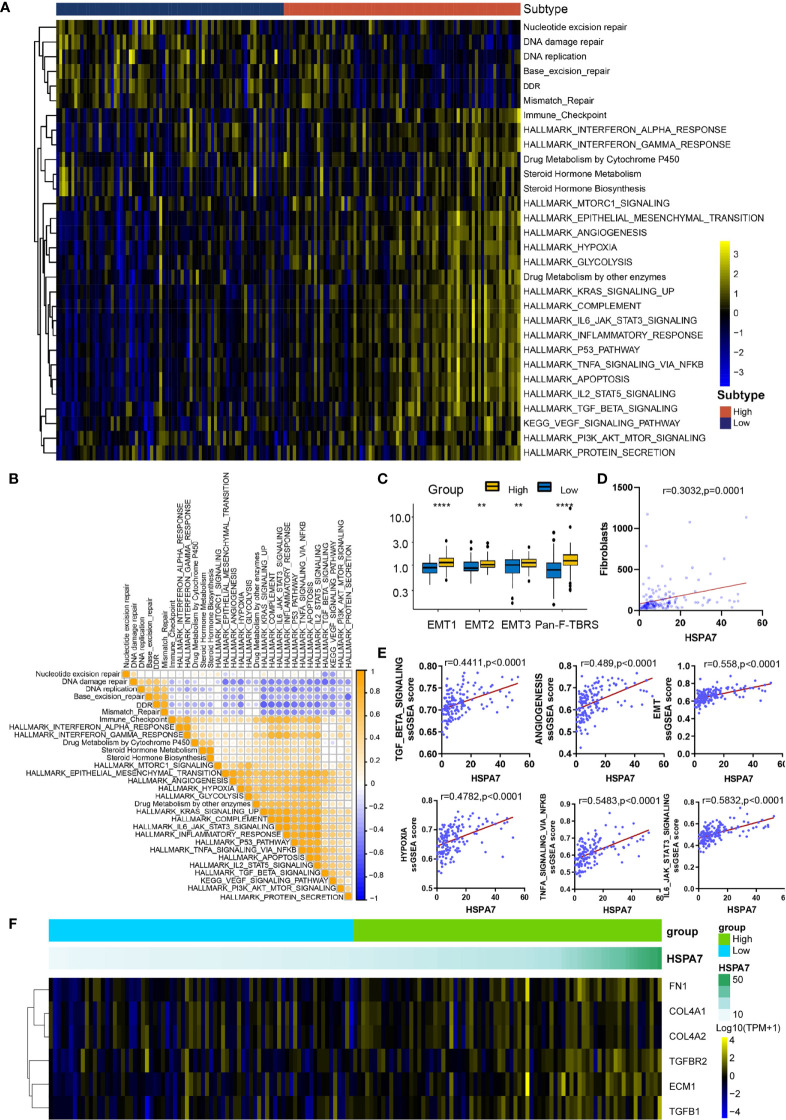
HSPA7 is correlated with stromal and carcinogenic activation pathways. **(A)** GSVA enrichment analysis showing the activation status of biological pathways in the HSPA7-high and HSPA7-low groups. A heatmap was used to visualize these biological processes. Yellow represents activated pathways, black represents moderately activated pathways, and blue represents inhibited pathways. **(B)** Correlations between each known gene signature in the TCGA GBM cohort using Pearson correlation analysis. Negative correlations are marked with blue, and positive correlations are marked with orange. **(C)** Differences in stromal activation-related pathways between the HSPA7-high and HSPA7-low groups. EMT, epithelial–mesenchymal transition; Pan-FTBRS, panfibroblast TGF-*β* response signature. **(D)** HSPA7 expression was positively correlated with fibroblast enrichment, as calculated by MCP-counter. **(E)** HSPA7 expression was positively correlated with stromal activation-related pathways. **(F)** A heatmap was used to visualize the expression of stromal activation-related genes. Yellow represents high expression, black represents the median expression, and blue represents low expression. The statistical significance is shown as: **P < 0.01; ****P < 0.0001.

To explore the differences in biological behaviors among these samples with distinct HSPA7 expression levels, we used the GSVA algorithm to estimate pathway enrichment scores for each sample (see *Materials and Methods*). Compared to the low HSPA7 expression group, the high HSPA7 expression group exhibited marked enrichment of stromal activation pathways [angiogenesis, epithelial–mesenchymal transition (EMT), VEGF, and TGF-β signaling pathways], oncogenic signaling pathways (hypoxia, apoptosis, PI3K Akt MTOR signaling, glycolysis, KRAS signaling, and other pathways), and immune responses (IL6 Jak stat3 signaling, which exerts immunosuppressive effects on T cell function and mediates ICB resistance in cancers (35), inflammatory response; interferon response; complement; and other pathways). However, the high HSPA7 expression group exhibited lower enrichment in pathways related to DNA replication- and DNA damage response-related functions (**Figure 4A**). Previous studies demonstrated that activation of stromal cells in the TME can induce T cell suppression and oncogenic signaling pathway activation *via* complex cellular and biological reconfiguration mechanisms (36, 37), attenuating the tumor response to PD-L1 blockade (28). As shown in **Figure 4B**, stromal activation pathways were positively correlated with carcinogenic signaling pathways and immune suppression pathways but negatively correlated with DNA damage response and repair pathways in TCGA GBM samples. Further analyses of the activity of stroma-related pathways indicated that high HSPA7 expression was significantly associated with higher stromal activation signatures (**Figure 4C**), as constructed by Mariathasan et al. (28), positively correlated with fibroblast enrichment (**Figure 4D**), as calculated by the MCP-counter method (38), and positively correlated with typical stromal cell activation-related pathways (**Figure 4E**). Furthermore, TGF-*β* is a pleiotropic cytokine associated with poor prognosis in GBM, playing a protumorigenic role by promoting immunosuppression, angiogenesis, metastasis, mesenchymal transition, and fibroblast activation (28, 39–41), and has been proven to promote the progression of GBM *via* an autocrine signaling loop (42). In our analysis, a TGF-*β* ligand (TGFB1) and a TGF-*β* receptor (TGFBR2), two key regulators in stromal activation and EMT pathways (28), and other genes encoding ECM and matricellular proteins (COLA4A1, COLA4A2, ECM1, and FN1) (8), which form a barrier to lymphocyte infiltration, showed increased expression in the high-HSPA7 group compared to the low-HSPA7 group (**Figure 4F**). These results suggested that immune cells and stromal cells in the TME can cooperate to synergistically regulate the immunosuppressive microenvironment, thereby promoting tumor immune escape.

**Figure 5:**
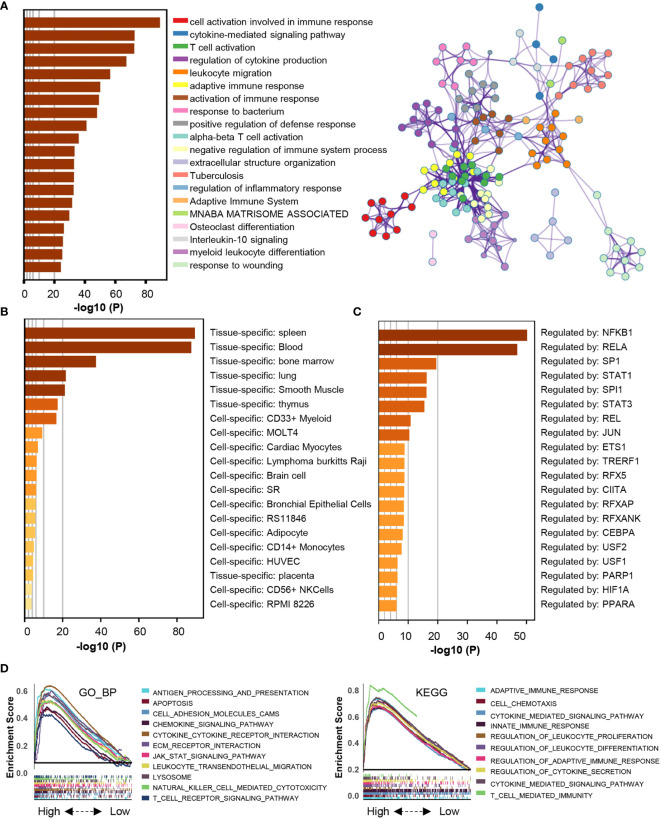
The genes that were positively correlated with HSPA7 were enriched in immune response- and stromal-related pathways. **(A)** Bar graph of enriched terms, colored by p-values, and corresponding network of enriched terms. **(B)** Summary of enrichment analysis in PaGenBase and **(C)** summary of enrichment analysis in TRRUST across HSPA7 positively correlated genes. **(D)** GSEA analyses displayed key immune-regulated pathways enriched in the high (up) and low (down) HSPA7 groups, both in the GO biological process (left) and KEGG datasets (right), and each line is for one pathway.

Then, we explored the pathways enriched with genes positively correlated with HSPA7 (Pearson’s r ≥ 0.3, P ≤ 0.05, **Table S7**) *via* the Metascape database (see *Materials and Methods*). These genes were significantly enriched in pathways related to the immune response, including cytokine-mediated signaling pathways, leukocyte migration, differentiation, and other immune-related pathways. Additionally, they were enriched in pathways involving stromal activation such as extracellular structure organization and response to wounding. These enriched pathways interacted with each other to form a protein–protein interaction network (**Figure 5A**). Furthermore, enrichment analysis in the PaGenBase (43) showed that these genes were almost completely specifically expressed in spleen, blood, bone marrow, and some other tissues with peripheral immune cell aggregation (**Figure 5B**), suggesting that HSPA7 can indeed promote the infiltration of immune cells into tumor tissues. In addition, enrichment analysis in the TF-target interaction database Transcriptional Regulatory Relationships Unraveled by Sentence-based Text mining (TRRUST) (44) showed that the genes were substantially transcriptionally regulated by NF-*κ*B1 and RELA, two important transcription factors involved in NF-*κ*B signaling pathways (**Figure 5C**). Further GSEA using the Gene Ontology (GO) and KEGG databases also showed that HSPA7 expression was positively correlated with the immune response and extracellular structure organization (**Figure 5D**).

### Verification of the Functions of HSPA7 in Two CGGA Cohorts

To further validate the function of HSPA7 in GBM, we explored its expression pattern in two CGGA RNA-seq cohorts. The expression of HSPA7 was highest in GBM (WHO IV) among the glioma specimens with three different WHO grades (**Supplementary Figures S6A**, **S7A**). Kaplan–Meier survival analysis showed that GBM patients in both cohorts with high HSPA7 expression had poor survival outcomes (**Supplementary Figures S6B**, **S7B**). Further GSVA enrichment analysis also showed that HSPA7 expression was correlated with the immunophenotype, stromal activation, and oncogenic pathway activation in GBM samples (**Supplementary Figures S6C**, **S7C**). These results confirmed that HSPA7 can be used as an independent prognostic risk biomarker in GBM patients. Moreover, HSPA7 can regulate the TME immune response and stromal activation, promoting malignant progression of GBM tumors.

### M^6^A-Modified HSPA7 Is Potentially Regulated by the Methyltransferase WTAP

To explore the potential molecular mechanism regulating HSPA7 RNA metabolism, we first screened HSPA7-interacting proteins *via* the NCBI database (**Table S8**). We verified that WTAP (45), a regulatory subunit of the m^6^A methyltransferase (46, 47), is required for the localization of the m6A methyltransferase into nuclear speckles as a putative HSPA7-binding regulator. To better map the association between WTAP and HSPA7, we explored the expression pattern in different grades of glioma in the TCGA, CGGA, Gravendeel and Rembrandt datasets. As shown in **Supplementary Figure S8A**, the expression of WTAP was highest in the WHO IV (GBM) group among glioma samples of three WHO grades and normal brain samples. Furthermore, overexpression of WTAP correlated with poor overall survival of GBM patients in all four datasets (**Supplementary Figure S8B**). Moreover, the expression of WTAP was positively correlated with the expression of HSPA7 in the TCGA GBM dataset (as calculated *via* the GEPIA database) and two CGGA GBM datasets (**Supplementary Figure S8C**). Further GSVA enrichment analysis showed that compared to the WTAP-low group, the WTAP-high group showed increased immune cell infiltration into the TME, stromal activation and oncogenic pathway activation, accompanied by higher immune and stromal scores and lower tumor purity, in the TCGA GBM cohort (**Supplementary Figure S8D**), CGGA GBM cohort 1 (**Supplementary Figure S9A**) and CGGA GBM cohort 2 (**Supplementary Figure S9B**). These patterns were the same as the patterns observed for HSPA7 expression. The above findings indicate that m^6^A methylation of HSPA7 is regulated by WTAP, although this conclusion and the functional mechanisms need further demonstration. Subsequent pathway and process enrichment analyses of HSPA7-interacting proteins showed that they can mediate many biological behaviors, such as mitotic cell cycle phase transition, neddylation, proteasome-mediated ubiquitin-dependent protein catabolism, HIF1*α* pathway activity, and other functions (**Supplementary Figure S10A**, **Table S8**). Then, we further explored the enriched terms across the RNA-binding proteins (RBPs) detected by crosslinking immunoprecipitation coupled with sequencing (CLIP-seq) from data deposited in the starBase database. These RBPs were enriched mainly in terms such as mRNA processing, nucleic acid transport, and RNA catabolic process (**Supplementary Figure S10B** and **Table S9**). However, the exact mechanism by which HSPA7 regulates the GBM TME requires further clarification.

### HSPA7 Holds Promise for Predicting the Therapeutic Response to ICB Therapy

**Figure 6:**
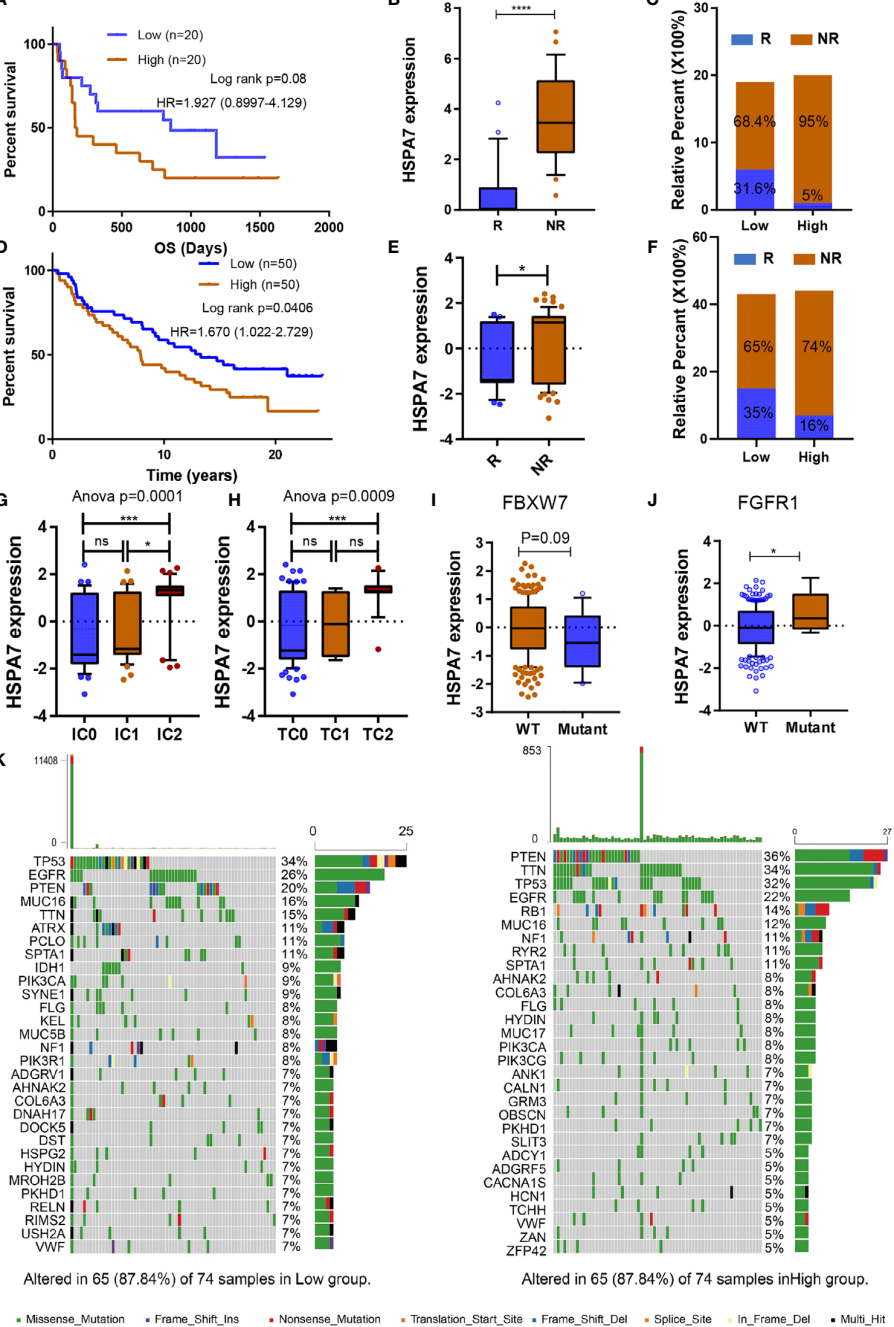
HSPA7 holds promise for predicting the therapeutic response to ICB. **(A)** The Kaplan–Meier survival curves showed that HSPA7 was a prognostic risk factor in the melanoma cohort that received anti-CTLA4 therapy. **(B)** Expression of HSPA7 in distinct anti-CTLA4 clinical response groups, R, response; NR, no response. **(C)** The proportion of patients who responded to CTLA4 blockade immunotherapy in the low or high HSPA7 expression groups. **(D)** The Kaplan–Meier survival curves showed that HSPA7 is a prognostic risk factor in the anti-PD-L1 cohort (IMvigor210). **(E)** Expression of HSPA7 in distinct anti-PD-L1 clinical response groups, R, response; NR, no response. **(F)** The proportion of patients who responded to PD-L1 blockade immunotherapy in the low or high HSPA7 expression groups. PD-L1 expression in both ICs **(G)** and TCs **(H)** was associated with HSPA7 expression, with the highest PD-L1 expression level in cells showing the highest HSPA7 expression. (ANOVA, p = 0.0001, p = 0.0009, respectively). HSAP7 expression between **(I)** FBXW7 wild-type (WT) and mutant status and **(J)** FGFR1 wild-type (WT) and mutant status. **(K)** The waterfall plot of tumor somatic mutations established by those with high HSPA7 expression (right) and low HSPA7 expression (left). The statistical significance is shown as: ns, P > 0.05; *P < 0.05; ***P < 0.001; ****P < 0.0001.

We desired to further investigate the capacity of HSPA7 to predict the response to immune checkpoint therapy in GBM. However, few published immunotherapy datasets include GBM patients who received ICB therapy. Thus, we used a cohort of melanoma patients who received anti-CTLA4 therapy (23) and urothelial cancer cohorts of patients who received anti-PD-1 therapy (IMvigor210) to perform a complementary evaluation of the ability of HSPA7 to predict the immunotherapy response. Patients in the anti-CTLA4 cohort with low HSPA7 expression exhibited significant clinical benefits [**Figure 6A**, anti-CTLA cohort, HR 1.927 (0.8997–4.129)]. Furthermore, compared to the HSPA7-high group, the HSPA7-low group exhibited a significant therapeutic benefit and clinical response to antiCTLA-4 immunotherapy (**Figures 6B, C**). Similar results were found in the anti-PD-L1 cohort. Kaplan–Meier analysis showed that the patients with the 50 lowest HSPA7 expression levels exhibited markedly prolonged survival compared to the patients with the 50 highest HSPA7 expression levels [**Figure 6D**, HR 1.670 (1.022–2.792)]. Additionally, the HSPA7-low group exhibited significantly better therapeutic and clinical responses to anti-PD-L1 immunotherapy than the HSPA7-high group (**Figures 6E, F**).

Further GSVA enrichment analysis showed that compared to the HSPA7-low group, the HSPA7-high group exhibited higher TME immune cell infiltration with higher immune and stromal scores, lower tumor purity (**Supplementary Figure S11A**), higher stromal activation, higher oncogenic pathway activation, and lower MMR pathway activation (**Supplementary Figure S11B**), completely consistent with the functional enrichment patterns in GBM (**Figures 3A**, **4A** and **Supplementary Figures S5C**, **S6C**). PD-L1 expression in immune cells (ICs) and tumor cells (TCs) was also assessed in the IMvigor210 cohort, and we examined the difference in HSPA7 expression among groups with different PD-L1 expression levels. As indicated in **Figures 6G, H**, patients with higher PD-L1 expression levels in either immune cells or tumor cells exhibited higher HSPA7 expression levels (ANOVA summary IC: P = 0.0001, TC: P = 0.0009), indicating that HSPA7 can upregulate PD-L1 expression, suppressing immune activation. Moreover, previous studies indicated that F-box and WD repeat domain containing 7 (FBXW7) is a vital tumor suppressor in various cancers, controlling proteasome-mediated degradation of oncoproteins such as cyclin E, c-Myc, Mcl-1, mTOR, Jun, and Notch (48) and that its loss-of-function mutation promotes resistance to anti-PD-1 therapy through downregulation of vital sensing pathways (49). Compared to the FBXW7 wild-type group, HSPA7 expression was obviously decreased in the FBXW7 mutant group (**Figure 6I**). Fibroblast growth factor receptor 1 (FGFR1) is frequently mutated in various tumors, and inhibitors of FGFR1 have shown promising therapeutic value in several preclinical models (50). Palakurthi S. et al. (51) found that the combination of FGFR inhibition and PD-1 blockade can promote tumor-intrinsic induction of antitumor immunity. We also found that HSPA7 expression was significantly higher in the FGFR mutant group than in the FGFR wild-type group (**Figure 6J**). Collectively, our work strongly indicates that HSPA7 expression contributes to predicting the response to immune checkpoint therapy.

### HSPA7 Facilitated Macrophage Infiltration and Could Be a Potential Immunotherapy Target for GBM Patients

**Figure 7:**
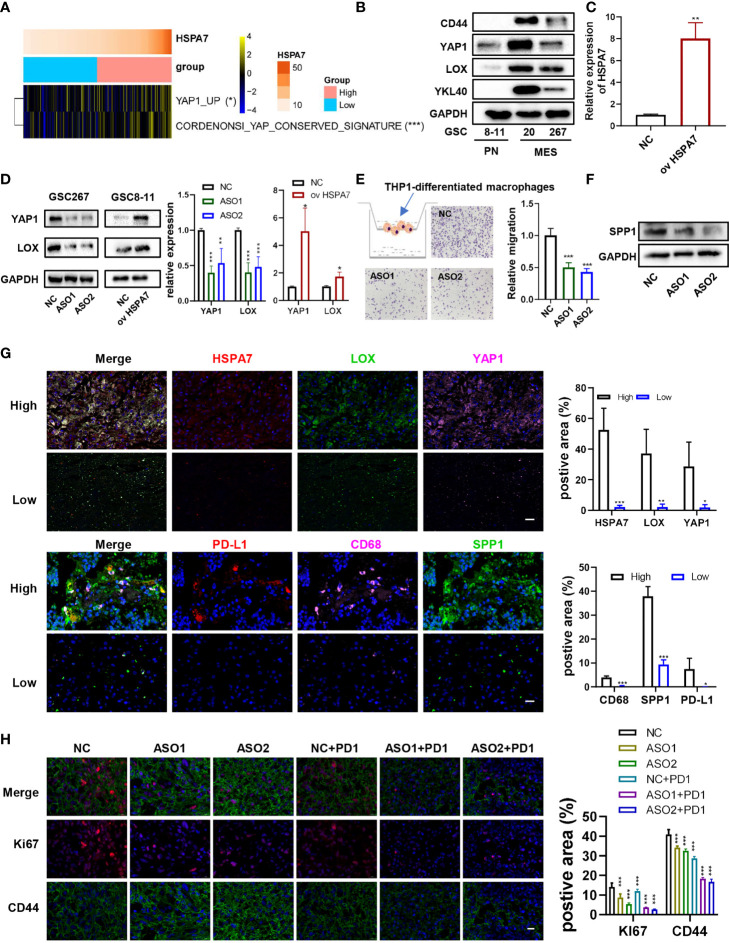
HSPA7 facilitated macrophage infiltration and might be a novel immunotherapy target for GBM patients. **(A)** The heatmap shows that HSPA7 activated YAP1 signaling. The asterisks indicate a significant statistical p-value calculated using the non-parametric Wilcoxon test (*P < 0.05; **P < 0.01; ***P < 0.001). **(B)** The protein expression of CD44, YAP1, LOX, and YKL40 in PN subtype GSCs (GSCs 8–11) and MES subtype GSCs (GSCs 20 and 267). **(C)** qPCR assays verified the overexpression efficiency of HSPA7. **(D)** Representative Western blot assays showed that HSPA7 promoted the expression of YAP1 and LOX, a macrophage chemoattractant (left), and quantification histogram represented the relative protein expression of LOX and YAP1 (right); data are presented as the mean ± SD, n = 3. *p < 0.05, **p < 0.01, ***p < 0.001. Means were compared with Student’s *t*-test for two groups and one-way ANOVA for three groups, and the NC group is indicated as the control. **(E)** Representative transwell migration assays showed that HSPA7 inhibited human THP1-differentiated macrophage migration by exposing them to conditioned medium from GSC 267 cells transfected with NC, HSPA7 ASO1, or ASO2 for 24 h. Original magnification, ×100 (left), and quantification histogram represented relative migration of THP-1 macrophages (right); data are presented as the mean ± SD, n = 3. *p < 0.05, **p < 0.01, ***p < 0.001. Means were compared with one-way ANOVA, and the NC group is indicated as the control. **(F)** Western blot assays showed that HSPA7 inhibited the expression of SPP1 in THP1-differentiated macrophages by exposing them to conditioned medium from GSC 267 cells transfected with NC, HSPA7 ASO1, or ASO2 for 48 h. **(G)** IF staining in a human GBM tissue microarray showed that the expression of LOX, YAP1, PD-L1, CD68, and SPP1 was higher in the HSPA7 high group than in the low group. Histogram representing statistical proportion data of positive area; data are presented as the mean ± SD, n = 3. *p < 0.05, **p < 0.01, ***p < 0.001, means were compared with Student’s *t*-test. **(H)** GBOs at 2 weeks were cocultured with conditioned medium from GSC 267 cells, transfected with NC, HSPA7 ASO1, or ASO2 at 48 h, and PD1 antibody (5 μM) for 5 days as indicated. IF staining for Ki67 and CD44 in GBO sections showed that knockdown of HSPA7 enhanced the effect of anti-PD1 therapy, original magnification, ×630 (scale bars: 20 μm). Histogram representing statistical proportion data of positive area; data are presented as the mean ± SD, n = 3. *p < 0.05, **p < 0.01, ***p < 0.001. Means were compared with one-way ANOVA, and the NC group is indicated as the control.

We then analyzed the differences in the distribution of somatic mutations between the low and high HSPA7 expression groups in the TCGA GBM cohort using the “maftools” package and found that the PTEN mutation rate was significantly increased in the HSPA7-high group compared to the HSPA7-low group (**Figure 6K**; low: 20%, high: 36%). Additionally, the NF1 mutation rate, which often occurs in the MES subtype and drives recruitment and activation of TAMs (33), was also obviously elevated in the HSPA7-high group compared to the HSPA7-low group (**Figure 6K**; low: 8%, high: 11%). Chen et al. (52) found that PTEN deficiency in GBM increases macrophage infiltration by activating YAP1 signaling, which directly upregulates lysyl oxidase (LOX) expression, an MES subtype marker and a macrophage chemoattractant (33), in turn inducing SPP1 secretion to support GBM survival. We found that YAP1 signaling was significantly upregulated in the HSPA7-high group compared to the HSPA7-low group (**Figure 7A**). We also confirmed that LOX and YAP1 were higher in MES GSCs than in PN GSCs (**Figure 7B**). Moreover, LOX and YAP1 expression levels were enhanced upon overexpression of HSPA7 in PN subtype GSCs 8–11 (**Figures 7C, D**) but decreased significantly upon knockdown of HSPA7 in MES subtype GSCs 267 (**Figure 7D**), suggesting that HSPA7 may facilitate tumor promoting macrophage infiltration by enhancing LOX expression, which could promote macrophage migration into the GBM TME and enhance angiogenesis by upregulating TAM-derived SPP1 (52). Finally, to further confirm the role of HSPA7 in facilitating macrophage migration by enhancing the YAP1-LOX axis, using the transwell assay, we found that conditioned medium from HSPA7 knockdown GSCs 267 inhibited THP1-differentiated macrophage migration significantly compared to the NC group (**Figure 7E**). Then, THP1-differentiated macrophages were cultured in conditioned medium collected from GSCs 267 transfected with HSPA7 ASO or NC. As shown in **Figure 7F**, conditioned medium from HSPA7-knockdown GSCs inhibited SPP1 expression in macrophages compared to the NC group. The immunofluorescent staining of our local clinical tumor tissue section analyses also showed that the expression of YAP1, LOX, CD68 (human macrophage marker), SPP1, and PD-L1 was enhanced in HSPA7-high GBM tissues compared to HSPA7-low tissues (**Figure 7G**).

Moreover, Robert M. Samstein et al. (53) found that TMB predicted survival after immunotherapy across multiple cancer types (including GBM). Thus, we downloaded the mutation data of 82 GBM samples and then analyzed the differences in the distribution of somatic mutations between the samples with the 30 lowest TMBs and the 30 highest TMBs. The PTEN and NF1 mutation rates were significantly increased in low-TMB samples compared to the high-TMB samples, and the same results were found for the HSPA7 expression level (**Supplementary Figure S12A**). We further explored the association between the HSPA7 expression level and TMB. As shown in **Supplementary Figure S12B**, HSPA7 expression was statistically negatively correlated with TMB. In addition, Touat M. et al. (54) recently found that mismatch repair (MMR)-deficient gliomas were characterized by a lack of prominent T cell infiltration, extensive intratumoral heterogeneity, poor patient survival, and a low rate of response to PD-1 blockade therapy. Our data also showed that the high HSPA7 expression group showed reduced activation of MMR-associated pathways (**Figure 4A**), which was negatively correlated with immunosuppression, stromal activation, and oncogene pathway activation (**Figure 4B**). Other studies have also shown the same results (28, 55). Furthermore, HSPA7 expression was negatively correlated with the expression of MMR genes such as MLH1, MSH2, MSH6, and PMS2 (**Supplementary Figure S12C**). Previous studies indicated that GBM patients exhibit resistance to immune checkpoint inhibitors (ICIs) due to the low mutation rate, the PTEN-deficient immunosuppressive microenvironment, infiltration by myeloid-derived suppressor cells (including TAMs, prominent players in brain cancers), and activation of tumor stromal cells (56, 57), indicating that knockdown of HSPA7 may enhance the efficacy of ICIs such as PD1 inhibitors in GBM. To illustrate this function, we generated patient-derived glioblastoma organoids (GBOs), which maintain cell-type heterogeneity (including macrophages, T cells, and vascular cells) and molecular signatures of their respective parental tumors, according to experimental protocols already reported by Jacob F. et al. (31). Immunofluorescence assays of our GBO sections also confirmed the presence of macrophages and vascular cells, as detected by CD68 and CD31 markers, respectively (**Supplementary Figure S13A**), supporting the use of three-dimensional GBO models for holistic study of the TME of GBM and evaluating the efficacy of PD1 inhibitors. We next applied our GBO model to test treatment responses *in vitro*. To mimic the postsurgical standard of care treatment, we subjected GBO samples from patient whose GBM tissues expressed high CD68 and SPP1 (**Supplementary Figure S13B**) to a single exposure conditioned medium from HSPA7-knockdown GSCs or with PD1 inhibitor (5 µM) treatment for 5 days. The therapeutic response was evaluated by quantifying the percentage of cells expressing Ki67 (proliferation index) and CD44 (invasion index). As shown in **Figure 7H**, knockdown of HSPA7 significantly enhanced the efficacy of the PD1 inhibitor in GBM. In summary, HSPA7 facilitated tumor promoting macrophage migration into the GBM TME by activating the YAP1–LOX axis, and our results indicated that HSPA7 might be a potential immunotherapy target for GBM patients.

### Characterization of HSPA7 Across 33 Cancer Types

We investigated whether HSPA7 expression is correlated with prognosis in a pancancer patient cohort. The expression of HSPA7 in tumor tissues and GTEx normal brain tissues was determined in the GEPIA database. As shown in **Supplementary Figure S14**, the expression of HSPA7 was significantly lower in tumor tissues in the ACC, COAD, DLBC, LAML, LUAD, LUSC, READ, THCA, and THYM cohorts but significantly higher in tumor tissues in the GBM, KIRC, KIRP, and PAAD cohorts than in the corresponding normal tissues. Genetic alterations were also observed in many other tumors, although these alterations were not significant. In addition, Cox regression analysis of survival rates and Kaplan–Meier analysis were conducted using the SangerBox tool. The relationships between HSPA7 expression levels and the prognoses of different tumors are shown in **Supplementary Figures S15**-**S17**. Statistically, HSPA7 is a risk factor in most cancers (such as ACC, LDBC, and CESC). However, we found that it is a relatively favorable factor in several cancer types such as SKCM, KIRP, and SARC. We next evaluated the immune score and stromal score across 33 cancers in the TCGA database. HSPA7 expression was significantly positively correlated with the immune score (**Supplementary Figure S18**) and stromal score (**Supplementary Figure S19**) in all 33 cancer types. Moreover, HSPA7 was significantly positively correlated with immune checkpoint expression (**Supplementary Figure S20A**) and the immune response (**Supplementary Figure S20B**) in most cancer types such as GBM, LGG, OV, and LUAD. However, no relationship was found in other cancers such as SKCM, UVM, TGCT, and PAAD, indicating that HSPA7 may act *via* a mechanism other than TME regulation in these cancers. Thus, the molecular mechanisms of this gene need further study in specific tumors. Collectively, these data emphasize that HSPA7 may be a key factor in facilitating the acquisition of various immunophenotypes in various cancers and may be considered in the development of more effective immunotherapies for GBM and other immunotherapy-resistant tumors. Because different immune cells may play different roles in different tumors, the functions of HSPA7 in specific tumors must be explored.

## Discussion

GBM, one of the most aggressive brain tumors, currently has no effective and sufficient therapies due to its intratumoral heterogeneity and molecular plasticity. ICB therapy is being actively pursued as a promising treatment option for GBM, but very few patients respond to ICB therapy. Thus, identifying markers regulating the brain TME that are prominent players in ICB therapy for cancer could reveal promising new targets for therapeutic intervention. LncRNAs and m^6^A modifications are emerging as indispensable regulators of the TME. However, the overall TME infiltration characteristics mediated by m^6^A-modified lncRNAs have not been comprehensively recognized. Therefore, it is worth obtaining comprehensive knowledge of the cellular TME infiltration characteristics mediated by m^6^A-regulated lncRNAs.

Here, based on our m^6^A-seq data, we revealed highly distinct lncRNA m^6^A methylation modification patterns between GBM and normal brain tissues. In addition, we identified immune-stromal-m^6^A-related HSPA7 as a novel prognostic risk factor in GBM patients; this gene plays a crucial role in immunophenotype determination, stromal activation, and oncogenic pathway activation and has a robust capacity to predict the ICB response. Furthermore, in this study, WTAP, a methyltransferase mediating m^6^A modification, was identified as a potential regulator of HSPA7 expression. Further analysis showed that WTAP, like HSPA7, can also regulate immunophenotype determination and stromal activation-related pathways (**Supplementary Figures S8**, **S9**). This finding again demonstrated that m^6^A modification is highly important in shaping the TME landscape. Many studies have reported that stromal cell activation in the TME can suppress immune infiltration or facilitate an immunosuppressive response in the TME, mediating therapeutic resistance to ICB (28, 58). Our data also revealed a markedly negative correlation between HSPA7 expression and TMB, a marker of the response to ICB therapy, in a TCGA GBM cohort (**Figure 6B**). In addition, another study showed that PTEN mutations were associated with immunosuppressive expression signatures in non-responders to anti-PD-1 immunotherapy in GBM (2). Chen et al. (52) found that PTEN deficiency in GBM increases the infiltration of SPP1^+^ macrophages, which can interact with fibroblasts and vascular endothelial cells, inducing angiogenesis, EMT, and some other stromal activation-related pathways in colon cancer and are characterized by expression of the pattern recognition receptor MARCO (59) *via* a YAP1–LOX-*β*1 integrin–PYK2 axis. Intriguingly, antibody-mediated depletion of MARCO inhibits cancer progression and metastasis, enhancing ICB efficacy (60), suggesting that SPP1^+^ macrophages play a non-negligible role in the immunorepressive response and immunotherapeutic resistance. We then analyzed myeloid cell-derived macrophage-restricted chemokines, which were the main factors that cause immunosuppression in GBM. We found that compared to tumors with low HSPA7 expression, tumors with high HSPA7 expression exhibited significantly increased myeloid cell-derived macrophage-restricted chemokines (8, 25) (**Supplementary Figures S4D, E**); we also confirmed that HSPA7 could facilitate the macrophage infiltration into the GBM TME *via* YAP1–LOX axis *in vitro*, as well as into our local GBM tissue sections (**Figures 7A–G**). Furthermore, we found that HSPA7 can interact with YAP1 (61), which can induce the secretion of CCL2/CSF1 to recruit monocytes (62), suggesting that HSPA7 may be a target that synergistically regulates SPP1^+^ macrophages, which then induce stromal activation in the TME. Moreover, we found that HSPA7 and YAP are also contained in GBM extracellular exosomes (63), a fundamental regulator of TME cells. Numerous research reports have indicated that YAP is a hub in the network of signals exchanged within the TME (64), thus regulating the immune response and stromal activation. However, the specific mechanism of HSPA7 needs to be proven by a large number of experiments. We also confirmed the predictive value of HSPA7 in a cohort of melanoma patients who received anti-CTLA4 therapy and an IMvigor210 cohort of patients treated with anti-PD-L1 therapy. A statistically significant difference in HSPA7 expression was found between non-responders and responders. MMR deficiency has recently emerged as a beneficial indicator of the response to PD-1 blockade in patients with cancer (65, 66), whereas we found that HSPA7 was negatively correlated with MMR-related pathway activity (**Figure 4A**), which in turn was negatively correlated with stromal activation and oncogenic pathway activation (**Figure 4B** and **Supplementary Figure S11**). This pattern suggests that MMR deficiency is an unfavorable factor for the response to ICB therapy. Other studies have shown the same results (28, 55). By comprehensive analysis of data for 10,294 samples, a recent study showed that MMR-deficient gliomas are characterized by a lack of prominent T cell infiltration, extensive intratumoral heterogeneity, poor patient survival, and a low rate of response to PD-1 blockade (54), reconfirming the ability of HSPA7 to predict the response to immunotherapy. We also explored the function of HSPA7 in various cancer types. As shown in **Figures S13**-**S19**, HSPA7 expression had prognostic significance in most cancers; in addition, it can regulate the expression of immune checkpoint genes and the activity of immune response pathways and was positively correlated with the immune score and stromal score in the majority of tumor types. HSPA7 was found to be a risk factor in the cohort of melanoma patients who received anti-CTLA4 therapy (**Figures 6A–C**) but was found to be a beneficial prognostic factor in the TCGA SKCM cohort (23) (**Supplementary Figures S14**, **S16**), exhibiting no obvious correlation with immune checkpoint expression (**Supplementary Figure S20A**) or the immune response (**Supplementary Figure S20B**). The mechanisms underlying this discrepancy need future exploration.

In summary, *via* integrated analysis of our own m^6^A-seq data and public clinical data, we found highly distinct lncRNA m^6^A methylation modification patterns between GBM and normal brain tissues and determined that HSPA7 could be used to evaluate the prognosis of GBM patients, as well as the corresponding cellular TME infiltration and activation characteristics of individual patients, and could predict the clinical efficacy of anti-PD-1/-PD-L1 immunotherapy in patients. More importantly, this study offers several novel insights regarding cancer immunotherapy; for example, targeting m^6^A regulators to change the m^6^A modification patterns of key immune-regulating targets may reverse unfavorable cellular TME infiltration characteristics. This study contributes to future exploration of novel drug combination strategies or novel immunotherapeutic agents.

## Data Availability Statement

The datasets presented in this study can be found in online repositories. The names of the repository/repositories and accession number(s) can be found below: https://www.ncbi.nlm.nih.gov/, SRA PRJNA661159.

## Ethics Statement

The studies involving human participants were reviewed and approved by Ethical Committee on Scientific Research of Shandong University Qilu Hospital (approval number: KYLL-2018-324). The patients/participants provided their written informed consent to participate in this study.

## Author Contributions

GL and HX supervised the project. RRZ designed the research and performed all experiments. BYL completed the basic experiment part. ZH performed statistical analysis with the R language. SJZ, QDG, PZ, WQ, SBW and ZHC were responsible for clinical sample collection and subsequent sample delivery. XG, YHQ, ZWP helped to revise the manuscript. All authors contributed to the article and approved the submitted version.

## Funding

This work was supported by grants from the National Natural Science Foundation of China (Nos. 81874083; 82072776; 82072775; 81702468; 81802966; 81902540; 81874082; 81472353), Natural Science Foundation of Shandong Province of China (Nos. ZR2019BH057; ZR2020QH174), Key clinical Research project of Clinical Research Center of Shandong University (2020SDUCRCA011) and Taishan Pandeng Scholar Program of Shandong Province (No. tspd20210322).

## Conflict of Interest

The authors declare that the research was conducted in the absence of any commercial or financial relationships that could be construed as a potential conflict of interest.

## References

[1] Jackson C Lim M. Immunotherapy for Glioblastoma: Playing Chess, Not Checkers. Clin Cancer Res: Off J Am Assoc Cancer Res (2018) 24(17):4059–61. 10.1158/1078-0432.ccr-18-0491 29691293

[2] Zhao J Chen A Gartrell R Silverman A Aparicio L Chu T. Immune and Genomic Correlates of Response to Anti-PD-1 Immunotherapy in Glioblastoma. Nat Med (2019) 25(3):462–9. 10.1038/s41591-019-0349-y PMC6810613 30742119

[3] Daniel P Sabri S Chaddad A Meehan B Jean-Claude B Rak J. Temozolomide Induced Hypermutation in Glioma: Evolutionary Mechanisms and Therapeutic Opportunities. Front Oncol (2019) 9:41. 10.3389/fonc.2019.00041 30778375 PMC6369148

[4] Johanns T Miller C Dorward I Tsien C Chang E Perry A. Immunogenomics of Hypermutated Glioblastoma: A Patient With Germline POLE Deficiency Treated With Checkpoint Blockade Immunotherapy. Cancer Discovery (2016) 6(11):1230–6. 10.1158/2159-8290.cd-16-0575 PMC5140283 27683556

[5] Bouffet E Larouche V Campbell B Merico D de Borja R Aronson M. Immune Checkpoint Inhibition for Hypermutant Glioblastoma Multiforme Resulting From Germline Biallelic Mismatch Repair Deficiency. J Clin Oncol: Off J Am Soc Clin Oncol (2016) 34(19):2206–11. 10.1200/jco.2016.66.6552 27001570

[6] Gutmann D Kettenmann H. Microglia/Brain Macrophages as Central Drivers of Brain Tumor Pathobiology. Neuron (2019) 104(3):442–9. 10.1016/j.neuron.2019.08.028 PMC7288606 31697921

[7] Friebel E Kapolou K Unger S Núñez N Utz S Rushing E. Single-Cell Mapping of Human Brain Cancer Reveals Tumor-Specific Instruction of Tissue-Invading Leukocytes. Cell (2020) 181(7):1626–42.e20. 10.1016/j.cell.2020.04.055 32470397

[8] Klemm F Maas R Bowman R Kornete M Soukup K Nassiri S. Interrogation of the Microenvironmental Landscape in Brain Tumors Reveals Disease-Specific Alterations of Immune Cells. Cell (2020) 181(7):1643–60.e17. 10.1016/j.cell.2020.05.007 32470396 PMC8558904

[9] Deng X Su R Weng H Huang H Li Z Chen J. RNA N-Methyladenosine Modification in Cancers: Current Status and Perspectives. Cell Res (2018) 28(5):507–17. 10.1038/s41422-018-0034-6 PMC5951805 29686311

[10] Yang Y Hsu P Chen Y Yang Y. Dynamic Transcriptomic mA Decoration: Writers, Erasers, Readers and Functions in RNA Metabolism. Cell Res (2018) 28(6):616–24. 10.1038/s41422-018-0040-8 PMC5993786 29789545

[11] Xu C Yuan B He T Ding B Li S. Prognostic Values of YTHDF1 Regulated Negatively by Mir-3436 in Glioma. J Cell Mol Med (2020) 24(13):7538–49. 10.1111/jcmm.15382 PMC7339155 32449290

[12] Dong Z Cui H. The Emerging Roles of RNA Modifications in Glioblastoma. Cancers (2020) 12(3):736. 10.3390/cancers12030736 PMC7140112 32244981

[13] Chai R Wu F Wang Q Zhang S Zhang K Liu Y. mA RNA Methylation Regulators Contribute to Malignant Progression and Have Clinical Prognostic Impact in Gliomas. Aging (2019) 11(4):1204–25. 10.18632/aging.101829 PMC6402513 30810537

[14] Visvanathan A Patil V Arora A Hegde A Arivazhagan A Santosh V. Essential Role of METTL3-Mediated mA Modification in Glioma Stem-Like Cells Maintenance and Radioresistance. Oncogene (2018) 37(4):522–33. 10.1038/onc.2017.351 28991227

[15] Zhang S Zhao B Zhou A Lin K Zheng S Lu Z. mA Demethylase ALKBH5 Maintains Tumorigenicity of Glioblastoma Stem-Like Cells by Sustaining FOXM1 Expression and Cell Proliferation Program. Cancer Cell (2017) 31(4):591–606.e6. 10.1016/j.ccell.2017.02.013 28344040 PMC5427719

[16] Zheng Q Hou J Zhou Y Li Z Cao X. The RNA Helicase DDX46 Inhibits Innate Immunity by Entrapping mA-Demethylated Antiviral Transcripts in the Nucleus. Nat Immunol (2017) 18(10):1094–103. 10.1038/ni.3830 28846086

[17] Gao Y Vasic R Song Y Teng R Liu C Gbyli R. mA Modification Prevents Formation of Endogenous Double-Stranded RNAs and Deleterious Innate Immune Responses During Hematopoietic Development. Immunity (2020) 52(6):1007–21.e8. 10.1016/j.immuni.2020.05.003 32497523 PMC7408742

[18] Han D Liu J Chen C Dong L Liu Y Chang R. Anti-Tumour Immunity Controlled Through mRNA mA Methylation and YTHDF1 in Dendritic Cells. Nature (2019) 566(7743):270–4. 10.1038/s41586-019-0916-x PMC6522227 30728504

[19] Li H Tong J Zhu S Batista P Duffy E Zhao J. mA mRNA Methylation Controls T Cell Homeostasis by Targeting the IL-7/STAT5/SOCS Pathways. Nature (2017) 548(7667):338–42. 10.1038/nature23450 PMC5729908 28792938

[20] Patil D Chen C Pickering B Chow A Jackson C Guttman M. M(6)A RNA Methylation Promotes XIST-Mediated Transcriptional Repression. Nature (2016) 537(7620):369–73. 10.1038/nature19342 PMC5509218 27602518

[21] Ni W Yao S Zhou Y Liu Y Huang P Zhou A. Long Noncoding RNA GAS5 Inhibits Progression of Colorectal Cancer by Interacting With and Triggering YAP Phosphorylation and Degradation and is Negatively Regulated by the mA Reader YTHDF3. Mol Cancer (2019) 18(1):143. 10.1186/s12943-019-1079-y 31619268 PMC6794841

[22] Huang H Weng H Chen J. mA Modification in Coding and Non-Coding RNAs: Roles and Therapeutic Implications in Cancer. Cancer Cell (2020) 37(3):270–88. 10.1016/j.ccell.2020.02.004 PMC7141420 32183948

[23] Van Allen E Miao D Schilling B Shukla S Blank C Zimmer L. Genomic Correlates of Response to CTLA-4 Blockade in Metastatic Melanoma. Sci (New York NY) (2015) 350(6257):207–11. 10.1126/science.aad0095 PMC5054517 26359337

[24] Bindea G Mlecnik B Tosolini M Kirilovsky A Waldner M Obenauf A. Spatiotemporal Dynamics of Intratumoral Immune Cells Reveal the Immune Landscape in Human Cancer. Immunity (2013) 39(4):782–95. 10.1016/j.immuni.2013.10.003 24138885

[25] Bowman R Klemm F Akkari L Pyonteck S Sevenich L Quail D. Macrophage Ontogeny Underlies Differences in Tumor-Specific Education in Brain Malignancies. Cell Rep (2016) 17(9):2445–59. 10.1016/j.celrep.2016.10.052 PMC5450644 27840052

[26] Liberzon A Birger C Thorvaldsdóttir H Ghandi M Mesirov J Tamayo P. The Molecular Signatures Database (MSigDB) Hallmark Gene Set Collection. Cell Syst (2015) 1(6):417–25. 10.1016/j.cels.2015.12.004 PMC4707969 26771021

[27] Kanehisa M Sato Y Kawashima M Furumichi M Tanabe M. KEGG as a Reference Resource for Gene and Protein Annotation. Nucleic Acids Res (2016) 44:D457–62. 10.1093/nar/gkv1070 PMC4702792 26476454

[28] Mariathasan S Turley S Nickles D Castiglioni A Yuen K Wang Y. Tgfβ Attenuates Tumour Response to PD-L1 Blockade by Contributing to Exclusion of T Cells. Nature (2018) 554(7693):544–8. 10.1038/nature25501 PMC6028240 29443960

[29] Zeng D Ye Z Wu J Zhou R Fan X Wang G. Macrophage Correlates With Immunophenotype and Predicts Anti-PD-L1 Response of Urothelial Cancer. Theranostics (2020) 10(15):7002–14. 10.7150/thno.46176 PMC7295060 32550918

[30] Schalper K Rodriguez-Ruiz M Diez-Valle R López-Janeiro A Porciuncula A Idoate M. Neoadjuvant Nivolumab Modifies the Tumor Immune Microenvironment in Resectable Glioblastoma. Nat Med (2019) 25(3):470–6. 10.1038/s41591-018-0339-5 30742120

[31] Jacob F Salinas R Zhang D Nguyen P Schnoll J Wong S. A Patient-Derived Glioblastoma Organoid Model and Biobank Recapitulates Inter- and Intra-Tumoral Heterogeneity. Cell (2020) 180(1):188–204.e22. 10.1016/j.cell.2019.11.036 31883794 PMC7556703

[32] Hazra A Gogtay N. Biostatistics Series Module 3: Comparing Groups: Numerical Variables. Indian J Dermatol (2016) 61(3):251–60. 10.4103/0019-5154.182416 PMC4885176 27293244

[33] Wang Q Hu B Hu X Kim H Squatrito M Scarpace L. Tumor Evolution of Glioma-Intrinsic Gene Expression Subtypes Associates With Immunological Changes in the Microenvironment. Cancer Cell (2017) 32(1):42–56.e6. 10.1016/j.ccell.2017.06.003 28697342 PMC5599156

[34] Wu A Wei J Kong L Wang Y Priebe W Qiao W. Glioma Cancer Stem Cells Induce Immunosuppressive Macrophages/Microglia. Neuro-oncology (2010) 12(11):1113–25. 10.1093/neuonc/noq082 PMC3098021 20667896

[35] Tsukamoto H Fujieda K Miyashita A Fukushima S Ikeda T Kubo Y. Combined Blockade of IL6 and PD-1/PD-L1 Signaling Abrogates Mutual Regulation of Their Immunosuppressive Effects in the Tumor Microenvironment. Cancer Res (2018) 78(17):5011–22. 10.1158/0008-5472.can-18-0118 29967259

[36] Tomaszewski W Sanchez-Perez L Gajewski T Sampson J. Brain Tumor Microenvironment and Host State: Implications for Immunotherapy. Clin Cancer Res: Off J Am Assoc Cancer Res (2019) 25(14):4202–10. 10.1158/1078-0432.ccr-18-1627 PMC6635001 30804019

[37] Chen D Mellman I. Elements of Cancer Immunity and the Cancer-Immune Set Point. Nature (2017) 541(7637):321–30. 10.1038/nature21349 28102259

[38] Becht E Giraldo N Lacroix L Buttard B Elarouci N Petitprez F. Estimating The Population Abundance of Tissue-Infiltrating Immune and Stromal Cell Populations Using Gene Expression. Genome Biol (2016) 17(1):218. 10.1186/s13059-016-1070-5 27765066 PMC5073889

[39] Lin R Zhao L. Mechanistic Basis and Clinical Relevance of the Role of Transforming Growth Factor-β in Cancer. Cancer Biol Med (2015) 12(4):385–93. 10.7497/j.issn.2095-3941.2015.0015 PMC4706525 26779375

[40] Massagué J. TGFbeta in Cancer. Cell (2008) 134(2):215–30. 10.1016/j.cell.2008.07.001 PMC3512574 18662538

[41] Flavell R Sanjabi S Wrzesinski S Licona-Limón P. The Polarization of Immune Cells in the Tumour Environment by TGFbeta. Nat Rev Immunol (2010) 10(8):554–67. 10.1038/nri2808 PMC3885992 20616810

[42] Rodón L Gonzàlez-Juncà A Inda MM Sala-Hojman A Martínez-Sáez E Seoane J. Active CREB1 Promotes a Malignant Tgfβ2 Autocrine Loop in Glioblastoma. Cancer Discov (2014) 4(10):1230–41. 10.1158/2159-8290.cd-14-0275 25084773

[43] Pan J Hu S Shi D Cai M Li Y Zou Q. PaGenBase: A Pattern Gene Database for the Global and Dynamic Understanding of Gene Function. PloS One (2013) 8(12):e80747. 10.1371/journal.pone.0080747 24312499 PMC3846610

[44] Han H Cho J Lee S Yun A Kim H Bae D. TRRUST V2: An Expanded Reference Database of Human and Mouse Transcriptional Regulatory Interactions. Nucleic Acids Res (2018) 46:D380–D6. 10.1093/nar/gkx1013 PMC5753191 29087512

[45] Yue Y Liu J Cui X Cao J Luo G Zhang Z. VIRMA Mediates Preferential mA mRNA Methylation in 3’UTR and Near Stop Codon and Associates With Alternative Polyadenylation. Cell Discov (2018) 4:10. 10.1038/s41421-018-0019-0 29507755 PMC5826926

[46] Ping X Sun B Wang L Xiao W Yang X Wang W. Mammalian WTAP is a Regulatory Subunit of the RNA N6-Methyladenosine Methyltransferase. Cell Res (2014) 24(2):177–89. 10.1038/cr.2014.3 PMC3915904 24407421

[47] Liu J Yue Y Han D Wang X Fu Y Zhang L. A METTL3-METTL14 Complex Mediates Mammalian Nuclear RNA N6-Adenosine Methylation. Nat Chem Biol (2014) 10(2):93–5. 10.1038/nchembio.1432 PMC3911877 24316715

[48] Yeh C Bellon M Nicot C. FBXW7: A Critical Tumor Suppressor of Human Cancers. Mol Cancer (2018) 17(1):115. 10.1186/s12943-018-0857-2 30086763 PMC6081812

[49] Gstalder C Liu D Miao D Lutterbach B DeVine A Lin C. Inactivation of Fbxw7 of Impairs dsRNA Sensing and Confers Resistance to PD-1 Blockade. Cancer Discov (2020) 10(9):1296–311. 10.1158/2159-8290.cd-19-1416 PMC8802534 32371478

[50] Peng R Chen Y Wei L Li G Feng D Liu S. Resistance to FGFR1-Targeted Therapy Leads to Autophagy *via* TAK1/AMPK Activation in Gastric Cancer. Gastric Cancer: Off J Int Gastric Cancer Assoc Jpn Gastric Cancer Assoc (2020) 23(6):988–1002. 10.1007/s10120-020-01088-y 32617693

[51] Palakurthi S Kuraguchi M Zacharek S Zudaire E Huang W Bonal D. The Combined Effect of FGFR Inhibition and PD-1 Blockade Promotes Tumor-Intrinsic Induction of Antitumor Immunity. Cancer Immunol Res (2019) 7(9):1457–71. 10.1158/2326-6066.cir-18-0595 31331945

[52] Chen P Zhao D Li J Liang X Li J Chang A. Symbiotic Macrophage-Glioma Cell Interactions Reveal Synthetic Lethality in PTEN-Null Glioma. Cancer Cell (2019) 35(6):868–84.e6. 10.1016/j.ccell.2019.05.003 31185211 PMC6561349

[53] Samstein R Lee C Shoushtari A Hellmann M Shen R Janjigian Y. Tumor Mutational Load Predicts Survival After Immunotherapy Across Multiple Cancer Types. Nat Genet (2019) 51(2):202–6. 10.1038/s41588-018-0312-8 PMC6365097 30643254

[54] Touat M Li Y Boynton A Spurr L Iorgulescu J Bohrson C. Mechanisms and Therapeutic Implications of Hypermutation in Gliomas. Nature (2020) 580(7804):517–23. 10.1038/s41586-020-2209-9 PMC8235024 32322066

[55] Zhang B Wu Q Li B Wang D Wang L Zhou Y. mA Regulator-Mediated Methylation Modification Patterns and Tumor Microenvironment Infiltration Characterization in Gastric Cancer. Mol Cancer (2020) 19(1):53. 10.1186/s12943-020-01170-0 32164750 PMC7066851

[56] Jackson C Choi J Lim M. Mechanisms of Immunotherapy Resistance: Lessons From Glioblastoma. Nat Immunol (2019) 20(9):1100–9. 10.1038/s41590-019-0433-y 31358997

[57] Lim M Xia Y Bettegowda C Weller M. Current State of Immunotherapy for Glioblastoma. Nat Rev Clin Oncol (2018) 15(7):422–42. 10.1038/s41571-018-0003-5 29643471

[58] Afik R Zigmond E Vugman M Klepfish M Shimshoni E Pasmanik-Chor M. Tumor Macrophages are Pivotal Constructors of Tumor Collagenous Matrix. J Exp Med (2016) 213(11):2315–31. 10.1084/jem.20151193 PMC5068227 27697834

[59] Zhang L Li Z Skrzypczynska K Fang Q Zhang W O’Brien S. Single-Cell Analyses Inform Mechanisms of Myeloid-Targeted Therapies in Colon Cancer. Cell (2020) 181(2):442–59.e29. 10.1016/j.cell.2020.03.048 32302573

[60] Georgoudaki A Prokopec K Boura V Hellqvist E Sohn S Östling J. Reprogramming Tumor-Associated Macrophages by Antibody Targeting Inhibits Cancer Progression and Metastasis. Cell Rep (2016) 15(9):2000–11. 10.1016/j.celrep.2016.04.084 27210762

[61] Liu Y Zhang X Lin J Chen Y Qiao Y Guo S. CCT3 Acts Upstream of YAP and TFCP2 as a Potential Target and Tumour Biomarker in Liver Cancer. Cell Death Dis (2019) 10(9):644. 10.1038/s41419-019-1894-5 31501420 PMC6733791

[62] Zhang Y Zhang H Zhao B. Hippo Signaling in the Immune System. Trends Biochem Sci (2018) 43(2):77–80. 10.1016/j.tibs.2017.11.009 29249569

[63] Choi D Montermini L Kim D Meehan B Roth F Rak J. The Impact of Oncogenic EGFRvIII on the Proteome of Extracellular Vesicles Released From Glioblastoma Cells. Mol Cell Proteomics: MCP (2018) 17(10):1948–64. 10.1074/mcp.RA118.000644 PMC6166673 30006486

[64] Zanconato F Cordenonsi M Piccolo S. YAP and TAZ: A Signalling Hub of the Tumour Microenvironment. Nat Rev Cancer (2019) 19(8):454–64. 10.1038/s41568-019-0168-y 31270418

[65] Teo M Seier K Ostrovnaya I Regazzi A Kania B Moran M. Alterations in DNA Damage Response and Repair Genes as Potential Marker of Clinical Benefit From PD-1/PD-L1 Blockade in Advanced Urothelial Cancers. J Clin Oncol: Off J Am Soc Clin Oncol (2018) 36(17):1685–94. 10.1200/jco.2017.75.7740 PMC6366295 29489427

[66] Plimack E Dunbrack R Brennan T Andrake M Zhou Y Serebriiskii I. Defects in DNA Repair Genes Predict Response to Neoadjuvant Cisplatin-Based Chemotherapy in Muscle-Invasive Bladder Cancer. Eur Urol (2015) 68(6):959–67. 10.1016/j.eururo.2015.07.009 PMC4764095 26238431

