# Supplementary material for: The N6-Methyladenosine-Modified Pseudogene HSPA7 Correlates With the Tumor Microenvironment and Predicts the Response to Immune Checkpoint Therapy in Glioblastoma
Source: Front Immunol. 2021 Jul 20;12:653711. doi: 10.3389/fimmu.2021.653711 (PMC8329659; doi:10.3389/fimmu.2021.653711)
Supplement: Supplementary file 1 [file DataSheet_1.docx]

**Supplementary Results**


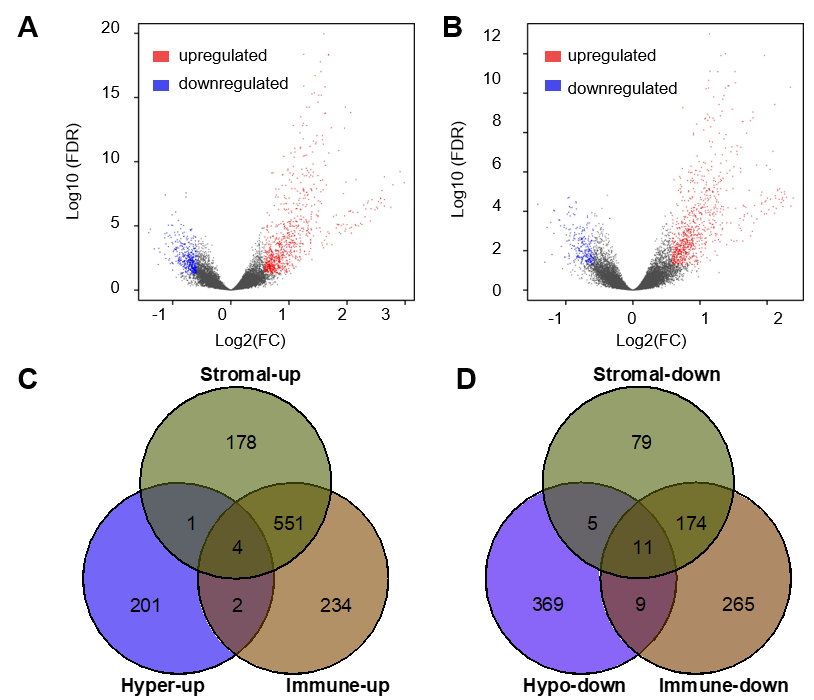


**Figure 1. Differentially expressed lncRNAs among immune， stromal and m^6^A regulated genes.** Volcano plot of the differential genes with **(A)** immune scores and **(B)** stromal scores of high score groups and low score groups in the median scores cutoff (|FC| ≥ 1.5, padj ≤ 0.05). Venn diagram of lncRNAs covered by **(C)** immune/stromal upregulated and m^6^A hyper-up lncRNAs, and **(D)** immune/stromal downregulated and m^6^A hypo-down lncRNAs.


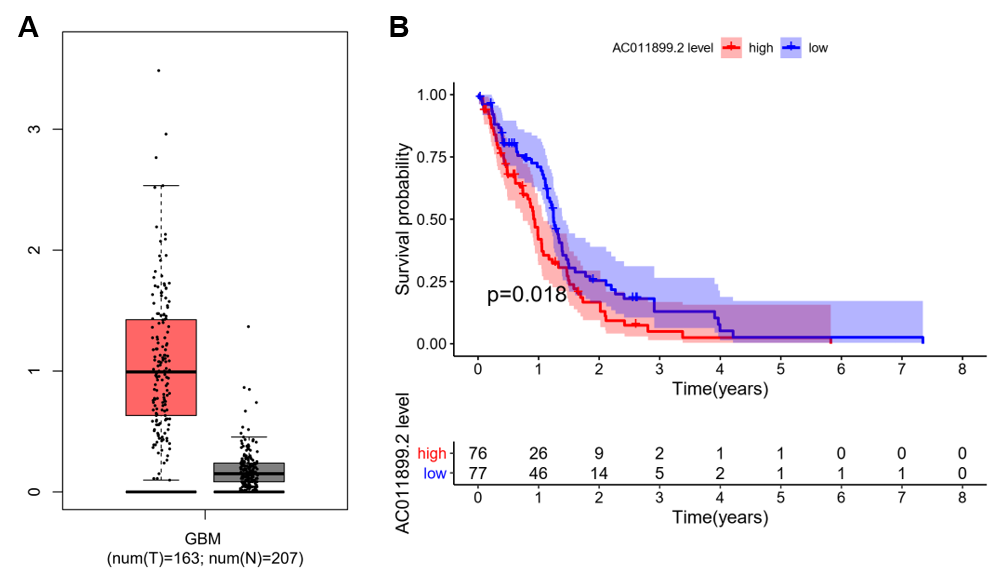


**Figure 2. AC011899.9 was not significantly overexpressed in GBM tissues, compared with GETx normal brain tissues. (A)** GEPIA database show that AC011899.9 was not over expressed significantly in GBM tissues, compared with GETx normal brain tissues. But **(B)** Even though the Kaplan–Meier survival curves show that AC011899.9 is a risk prognostic factor in GBM.


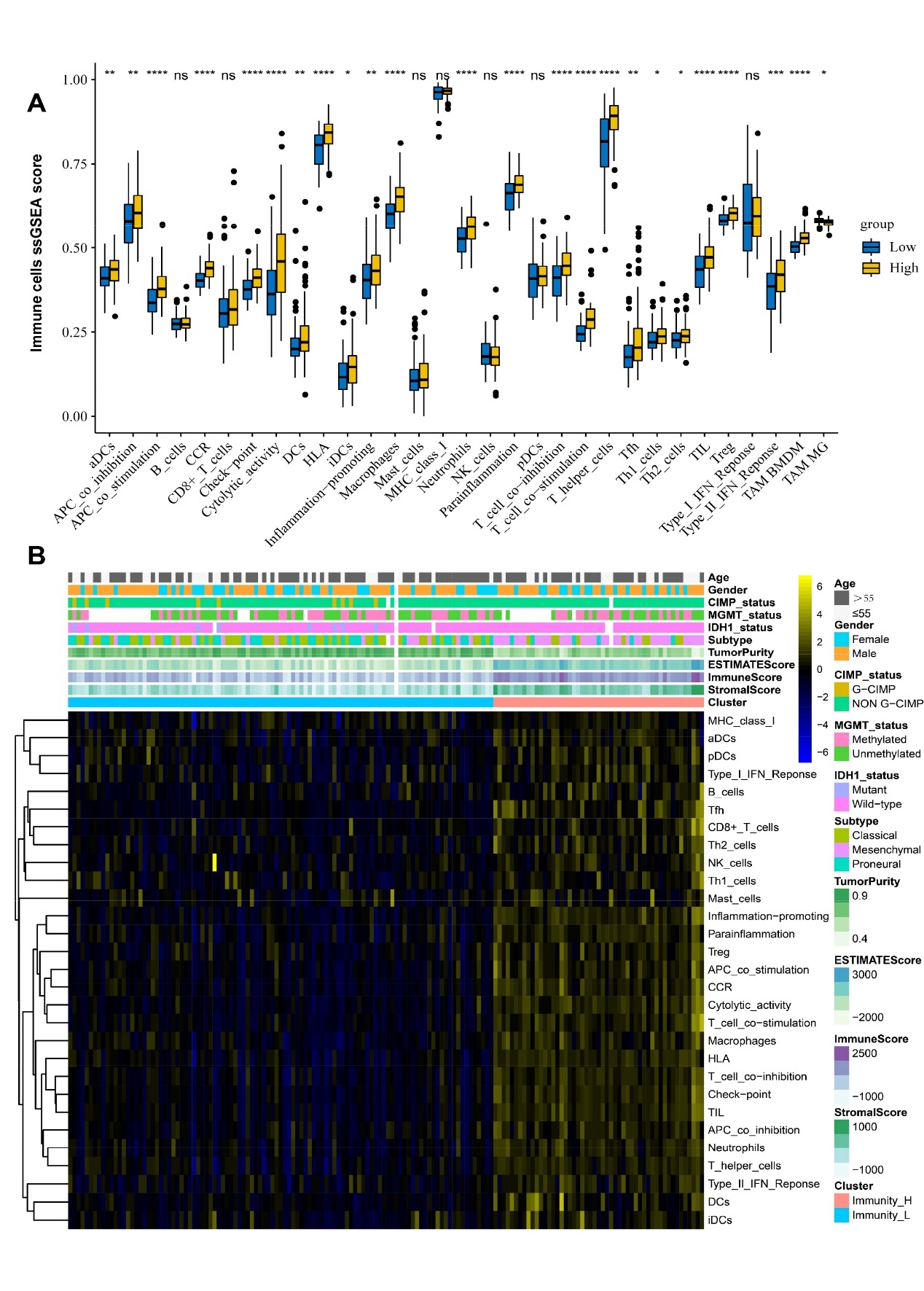


**Figure 3. TME landscapes of GBM in TCGA database. (A)** The abundance of each TME infiltrating cells and regulators in HSPA7 high and low groups. The asterisks indicated a significant statistical p-value calculated using the nonparametric Wilcoxon test (*P < 0.05; **P < 0.01; ***P < 0.001, ****P<0.0001). **(B)** Unsupervised clustering of TCGA GBM tissues using ssGSEA scores from immune related genesets.


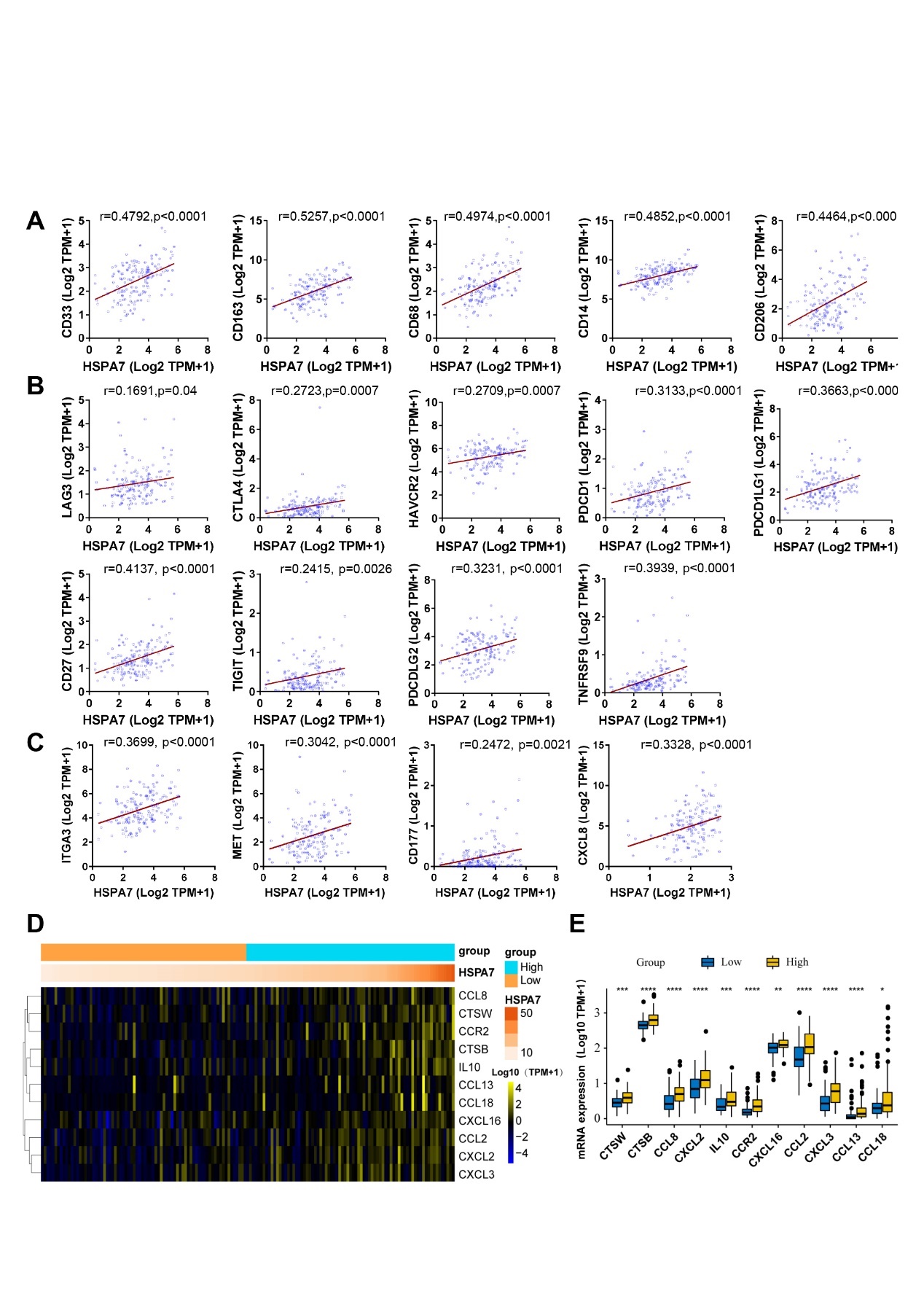


**Figure 4. HSPA7 upregulated the immune suppression genes.** HSPA7 had significantly positive correlation with the markers of **(A)** suppressor function of myeloid lineages, **(B)** immune inhibitory checkpoints and **(C)** major neutrophil-recruiting chemokines and their receptors. **(D)** The heatmap was used to visualize the anti-inflammatory genes of suppressor function of myeloid cells, yellow represented high expression, black represented median expression and blue represented low expression; **(E)** High expression of HSPA7 presented significantly increased anti-inflammatory regulators of suppressor function of myeloid cells. The asterisks indicate a significant statistical p-value calculated using the nonparametric Wilcoxon test (*P < 0.05; **P < 0.01; ***P < 0.001, ****P<0.0001).


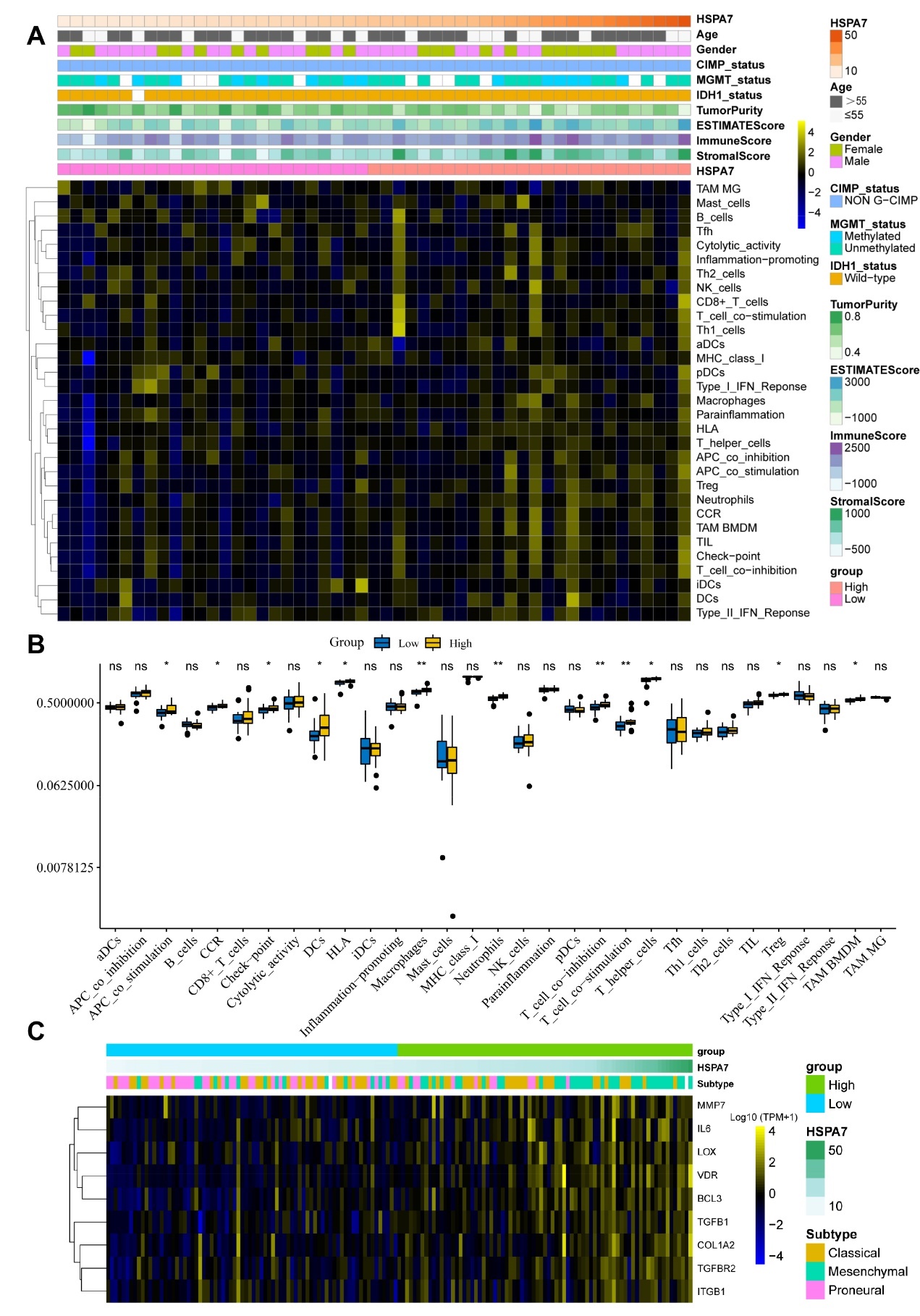


**Figure 5. HSPA7 activates the immune microenvironment by promoting the phenotypic transformation of GBM MES subtypes. (A)** The enrichment scores of immune cell types and immune related function related genesets in TCGA MES subtype GBM samples were calculated via ssGSEA algorithm. The heatmap was used to visualize these immune characteristics between HSAP7 High and Low expression group, and yellow represented high enrichment level, black represented median enrichment level and blue represented low enrichment level. **(B)** The abundance of each TME infiltrating cells and regulators in HSPA7 high and low groups. The asterisks indicated a significant statistical p-value calculated using the nonparametric Wilcoxon test (*P < 0.05; **P < 0.01; ***P < 0.001, ****P<0.0001). **(C)** The heatmap was used to visualize the expression of MES subtype signature genes, yellow represented high expression, black represented median expression and blue represented low expression.


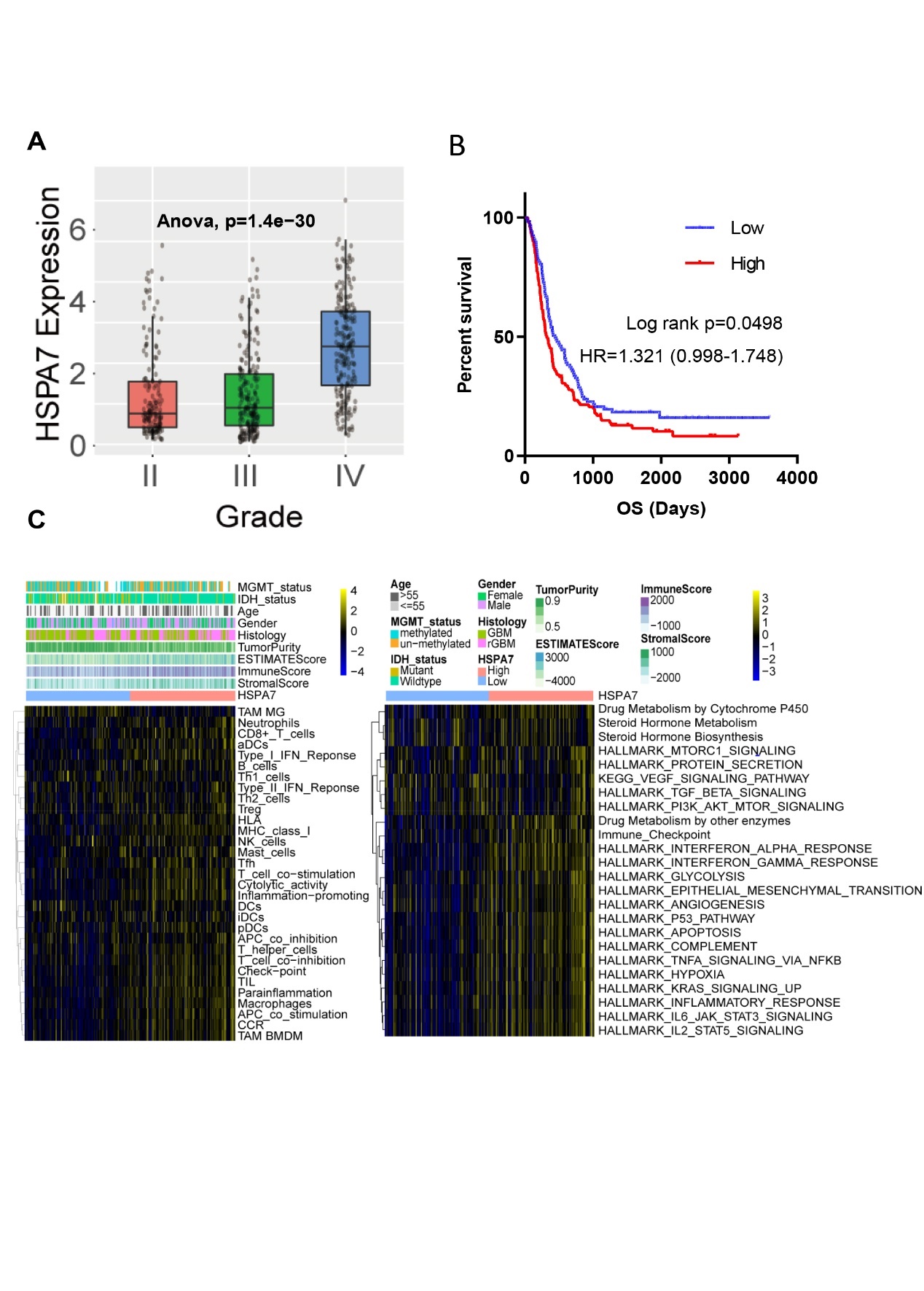


**Figure 6. The function of HSPA7 was verified in CGGA cohort 1.** **(A)** HSPA7 expression is the highest in GBM (WHO IV), compared to LGG samples (WHO II-III) in CGGA GBM cohort1 (n=237). **(B)** The Kaplan–Meier survival curves showed that HSPA7 is a risk prognostic factor in CGGA GBM cohort1. **(C)** GSVA enrichment analysis showing the immune cell infiltration, stromal and carcinogenic activation pathways enriched in HSPA7 high group, compared to the low group. The heatmap was used to visualize these biological processes, and yellow represented activated pathways, black represented median activated pathways and blue represented inhibited pathways. The following analysis were used as the same method.


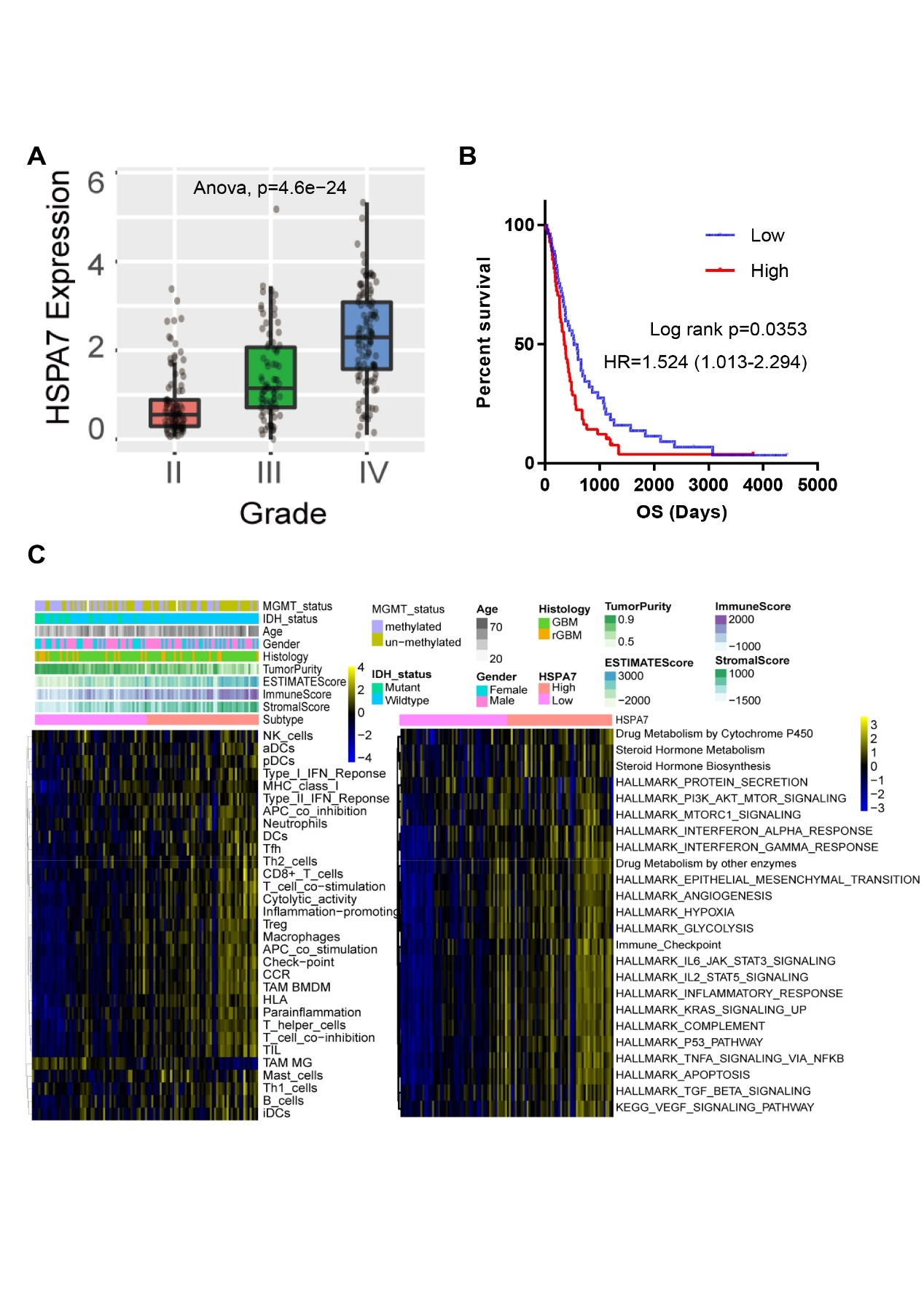


**Figure 7. The function of HSPA7 was verified in CGGA cohort 2.** **(A)** HSPA7 expression is the highest in GBM (WHO IV), compared to LGG samples (WHO II-III) in CGGA GBM cohort2 (n=108). **(B)** The Kaplan–Meier survival curves showed that HSPA7 is a risk prognostic factor in CGGA GBM cohort2. **(C)** GSVA enrichment analysis showing the immune cell infiltration, stromal and carcinogenic activation pathways enriched in HSPA7 high group, compared to the low group.


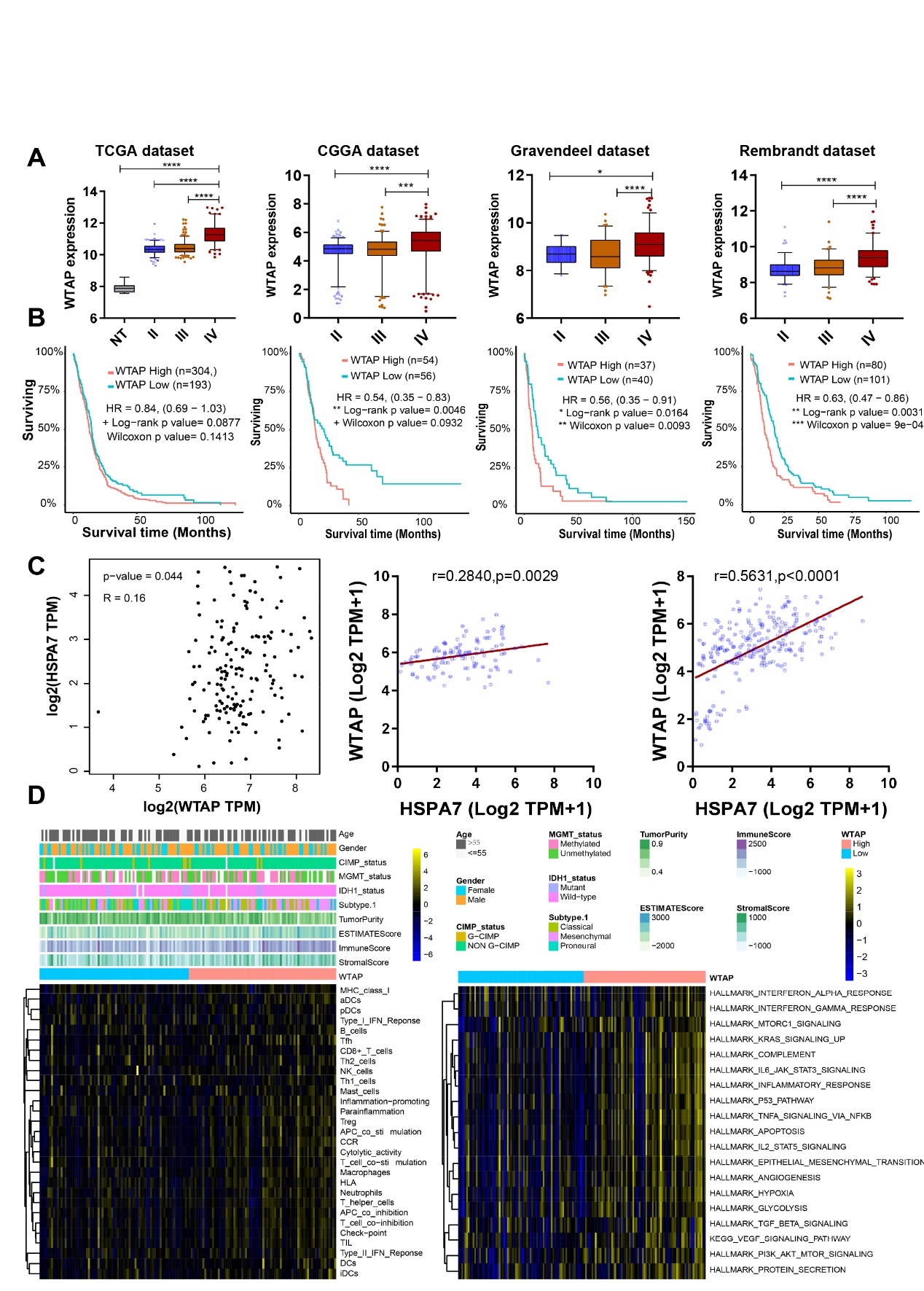


**Figure 8. WTAP potentially regulate recruitment of the m6A methyltransferase complex to HSAP7 directly. (A)** WTAP expression is the highest in GBM (WHO IV), compared to LGG samples (WHO II-III) or normal brain (NT) tissues in TCGA GBM, CGGA GBM, Gravendeel and Rembrandt cohorts. **(B)** The Kaplan–Meier survival curves showed that WTAP is a risk prognostic factor in TCGA GBM, CGGA GBM, Gravendeel and Rembrandt cohorts. **(C)** HSPA7 expression was correlated with WTAP expression in TCGA GBM and two CGGA GBM cohorts. **(D)** GSVA enrichment analysis showing the immune cell infiltration, stromal and carcinogenic activation pathways enriched in WTAP high group, compared to the low group in TCGA GBM cohort. The heatmap was used to visualize these biological processes, and yellow represented activated pathways, black represented median activated pathways and blue represented inhibited pathways.


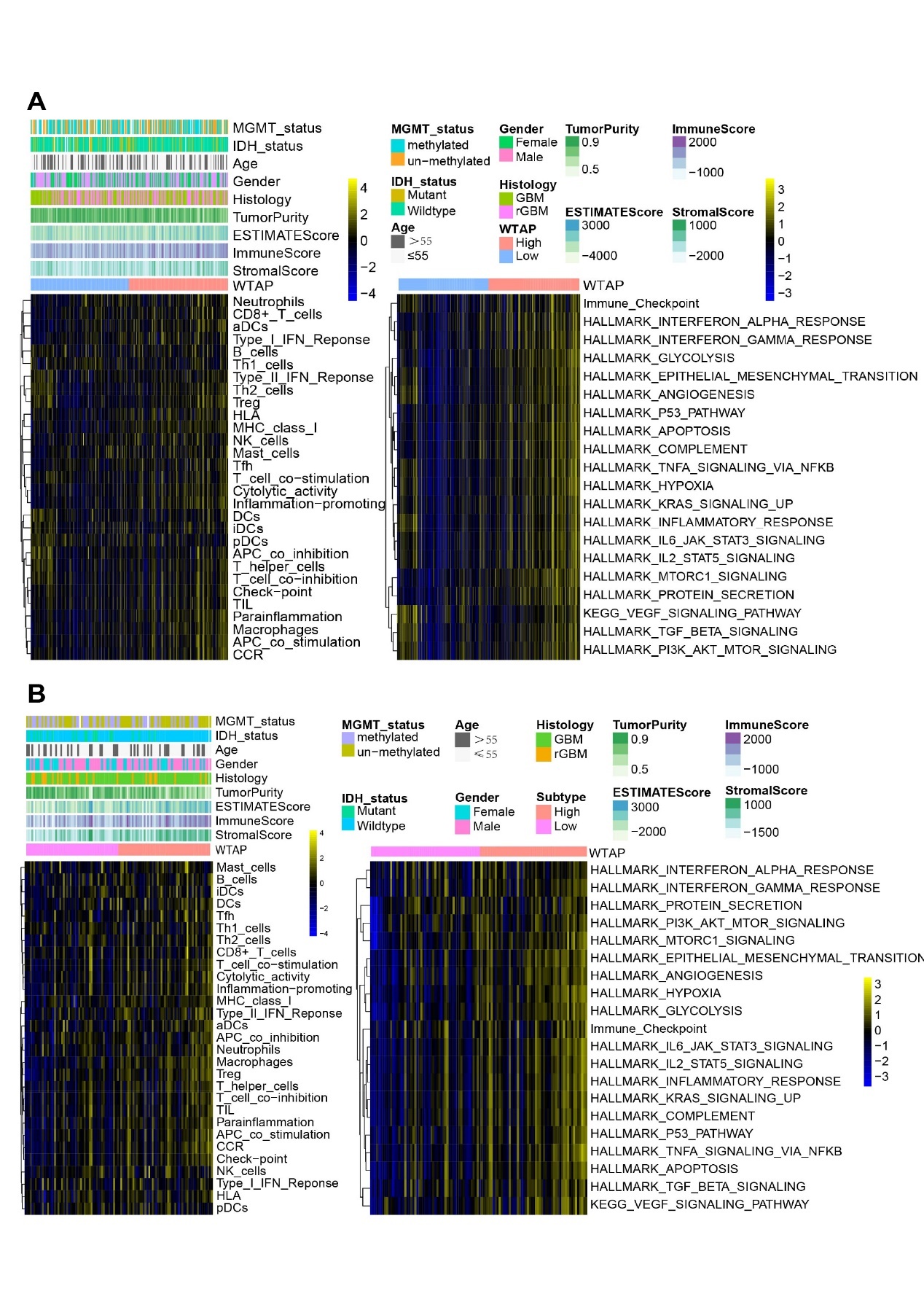


**Figure 9. WTAP correlated with immunophenotypes and stromal activation pathways in CGGA GBM datasets.** GSVA enrichment analysis showing the immune cell infiltration, stromal and carcinogenic activation pathways enriched in WTAP high group, compared to the low group in **(A)** CGGA GBM cohort1 (n=237) and **(B)** CGGA GBM cohort2 (n=108).


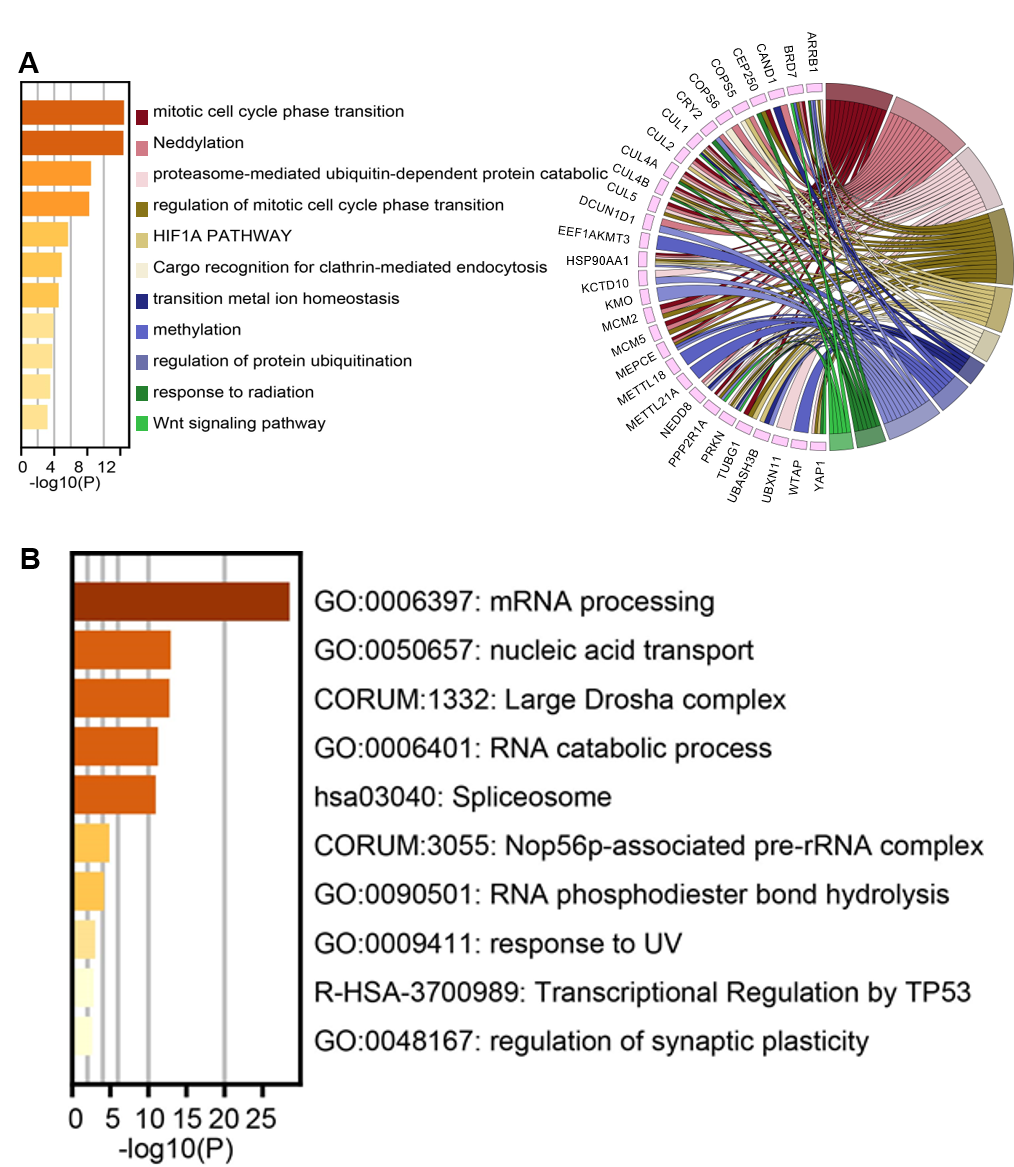


**Figure10. The enrichment of HSPA7 interacting proteins.** (A) Bar graph of enriched terms, colored by p-values, across HSPA7 interacting proteins identified in NCBI database, and the individual term enriched genes was visualized via circos string graphics (right). (B) Bar graph of enriched terms, colored by p-values, across HSPA7 interacting proteins identified in stabase database.


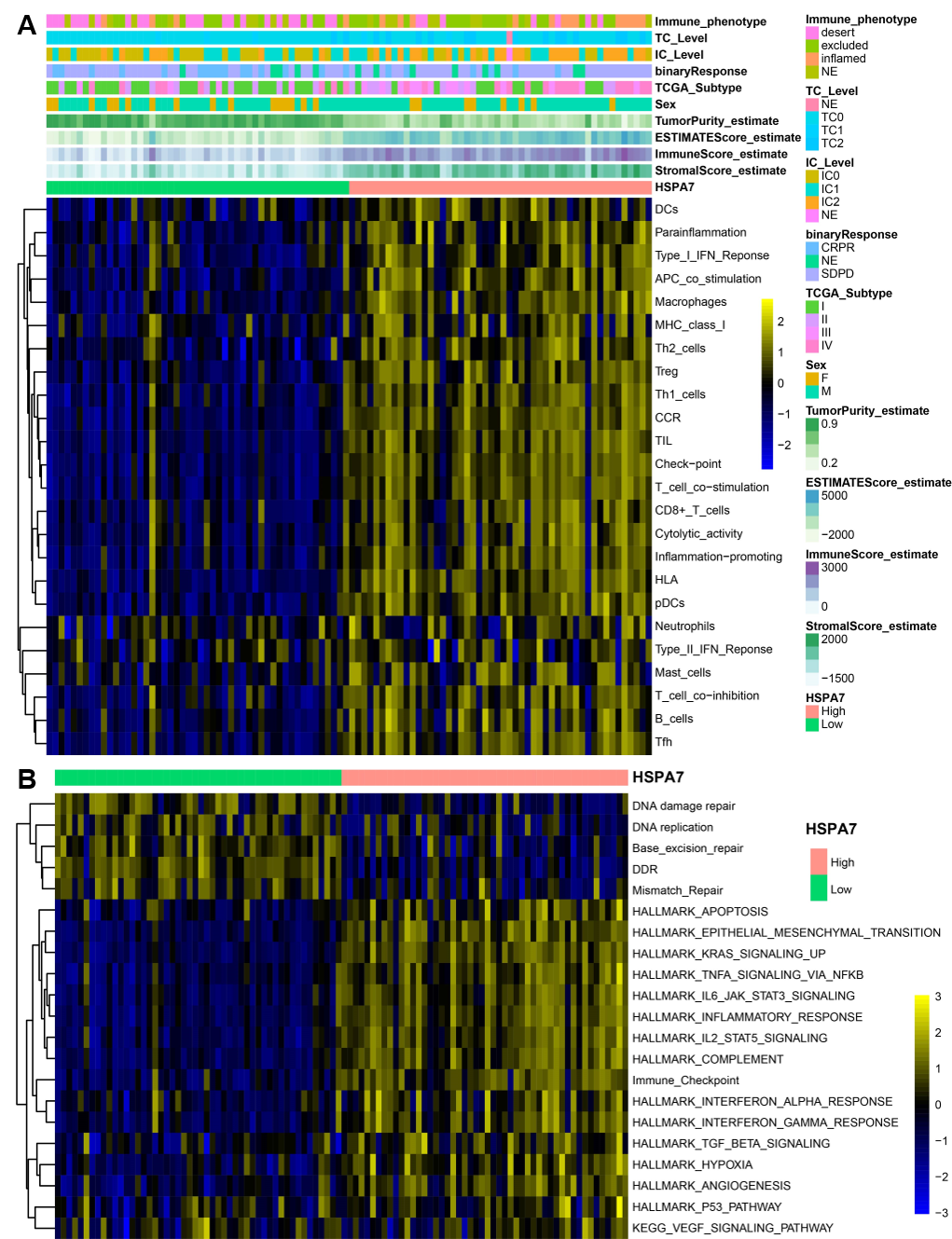


**Figure 11. HSPA7 correlated with the immunophenotypes and stomal activation in bladder cancers.** GSVA enrichment analysis showing **(A)** the immune cell infiltration, **(B)** stromal and carcinogenic activation pathways enriched in HSPA7 high group, compared to the low group in bladder cancers IMvigor210 cohort.


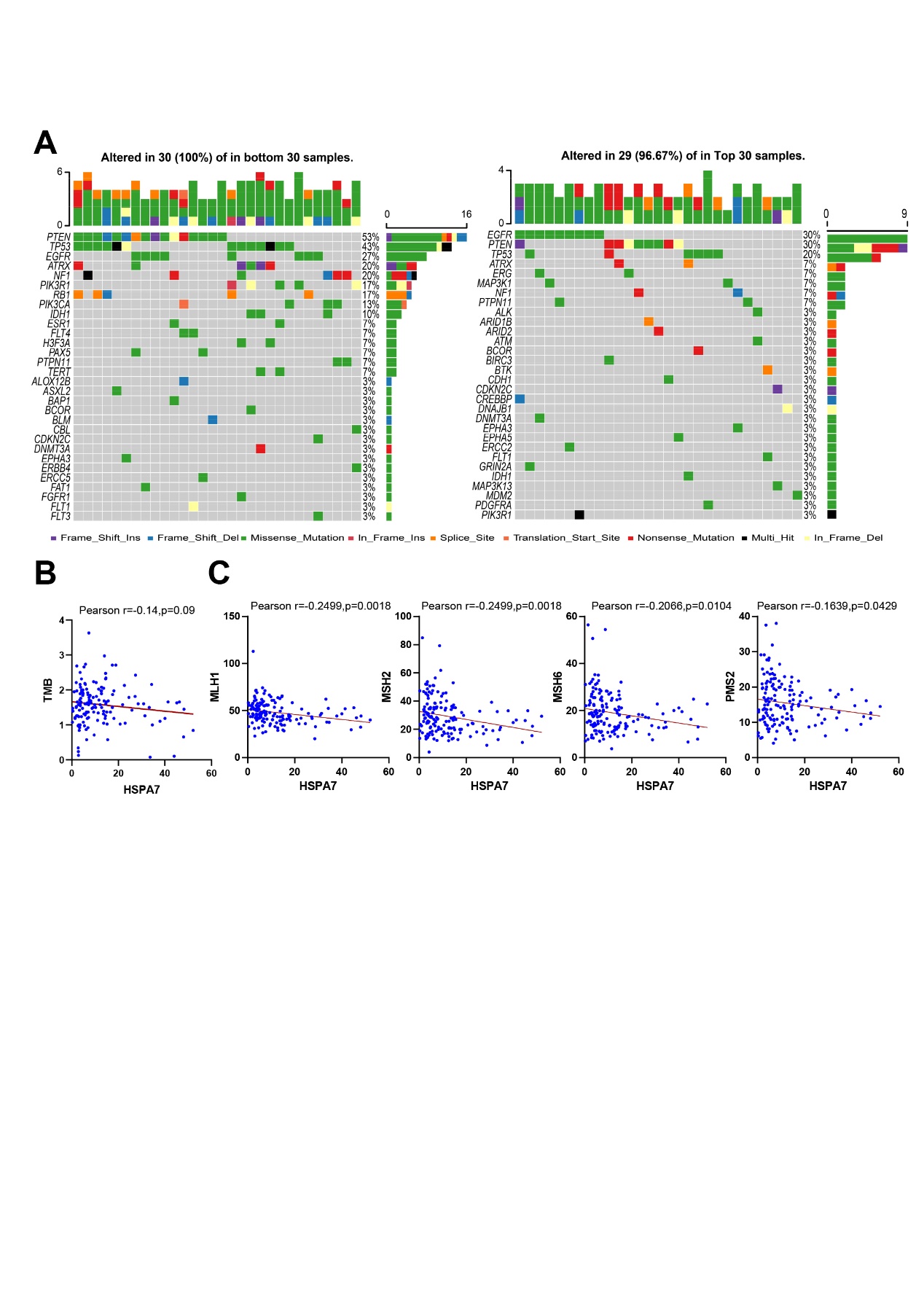


**Figure 12. HSPA7 negatively correlated with TMB and MMR genes. (A)** The waterfall plot of tumor somatic mutation established by those with top 30 high TMB group (right) and bottom 30 low group (left). HSPA7 negatively correlated with **(B)** TMB and **(C)** MMR genes (MLH1, MSH26, MSH6 and PMS2).


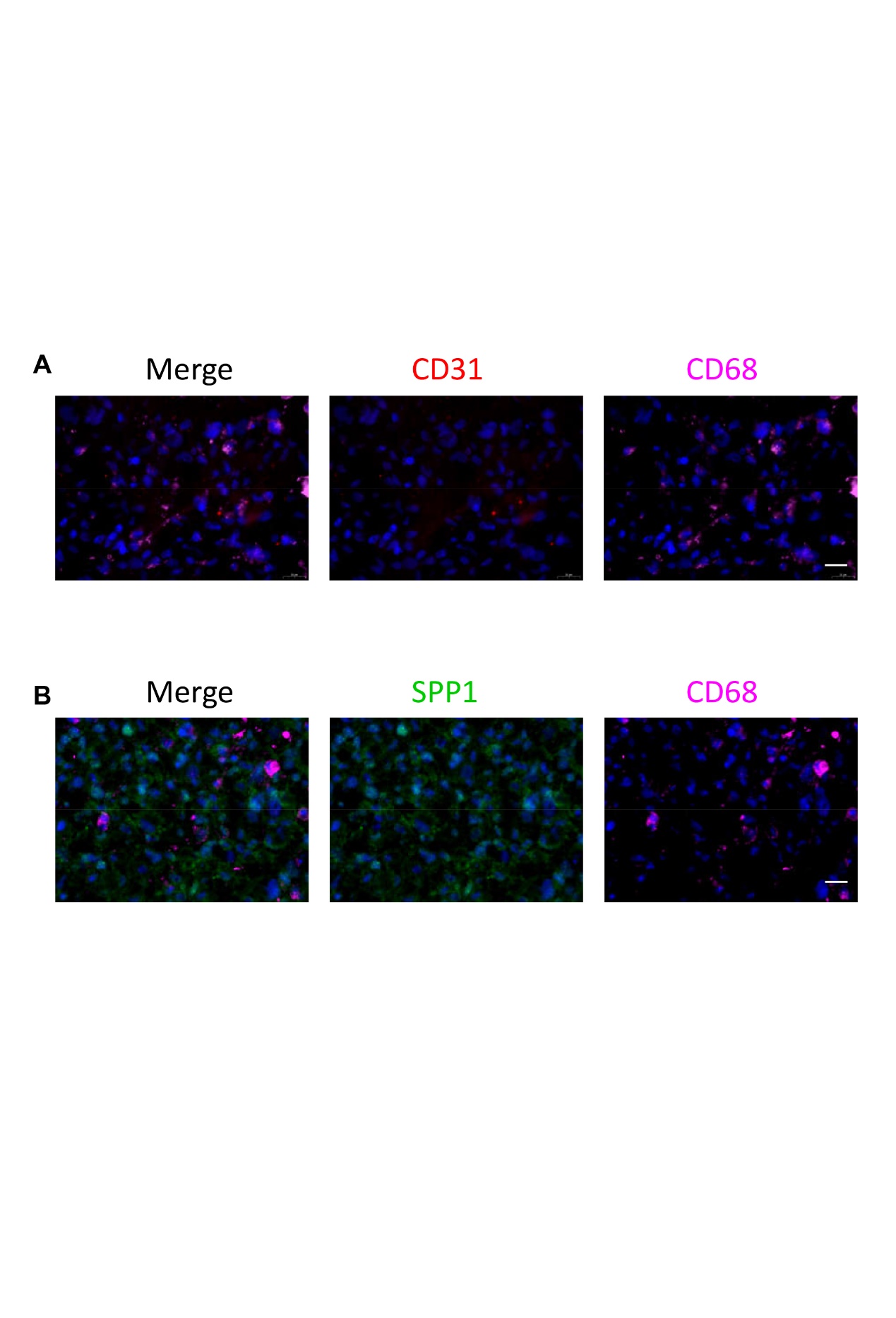


**Figure 13. GBOs were good models for holistic study the TME of GBM.** IF staining in human GBO tissue microarrays confirmed **(A)** the presence of macrophages and vascular cells, as detected by CD68 and CD31 markers respectively, and **(B)** the high expression of SPP1.


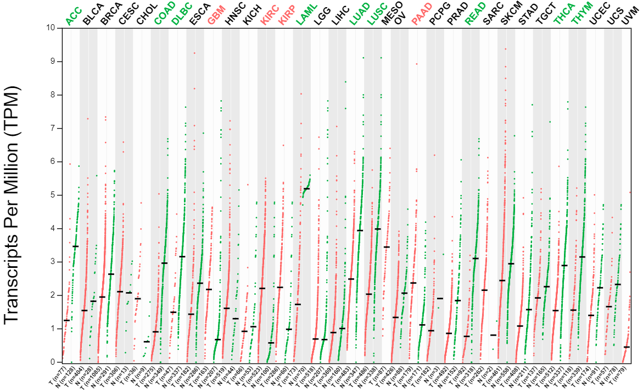


**Figure14 Expression changes of HSPA7 across 33 cancer types with their corresponding normal brain tissues.**


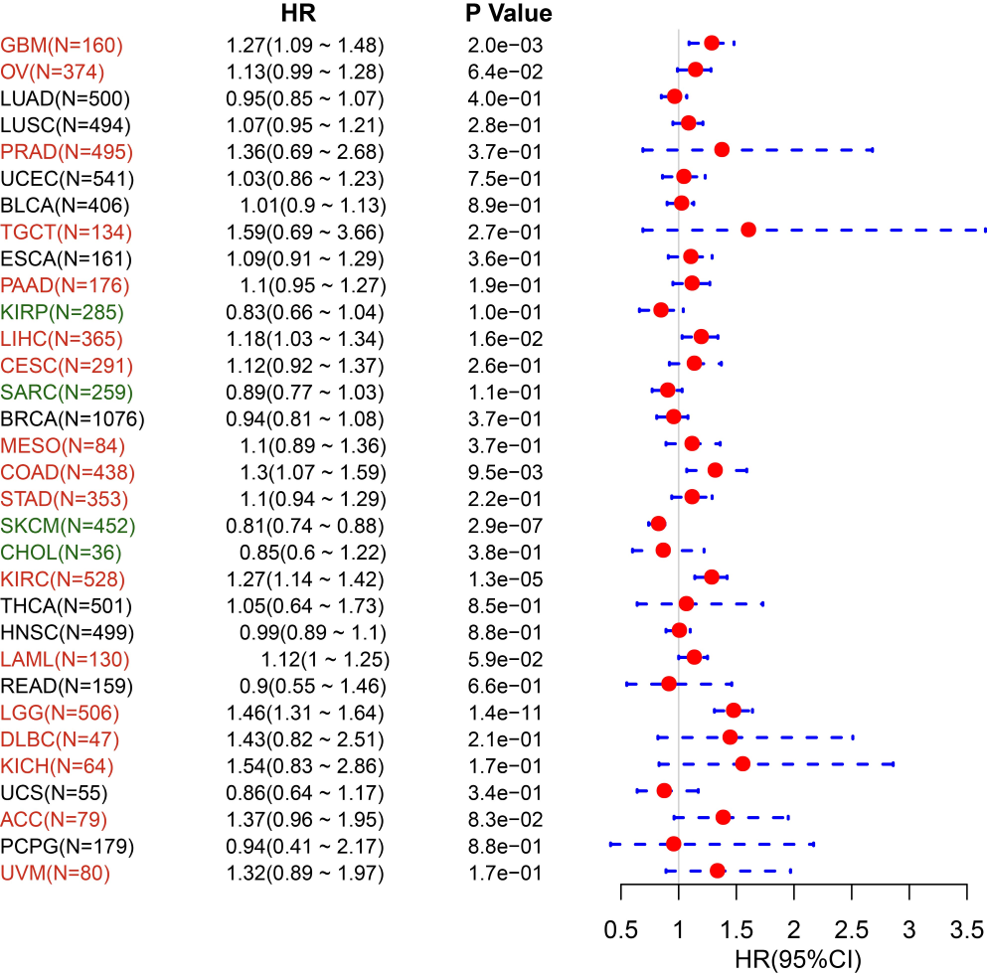


**Figure15 COX survival rates of HSPA7 across 33 cancer types.**


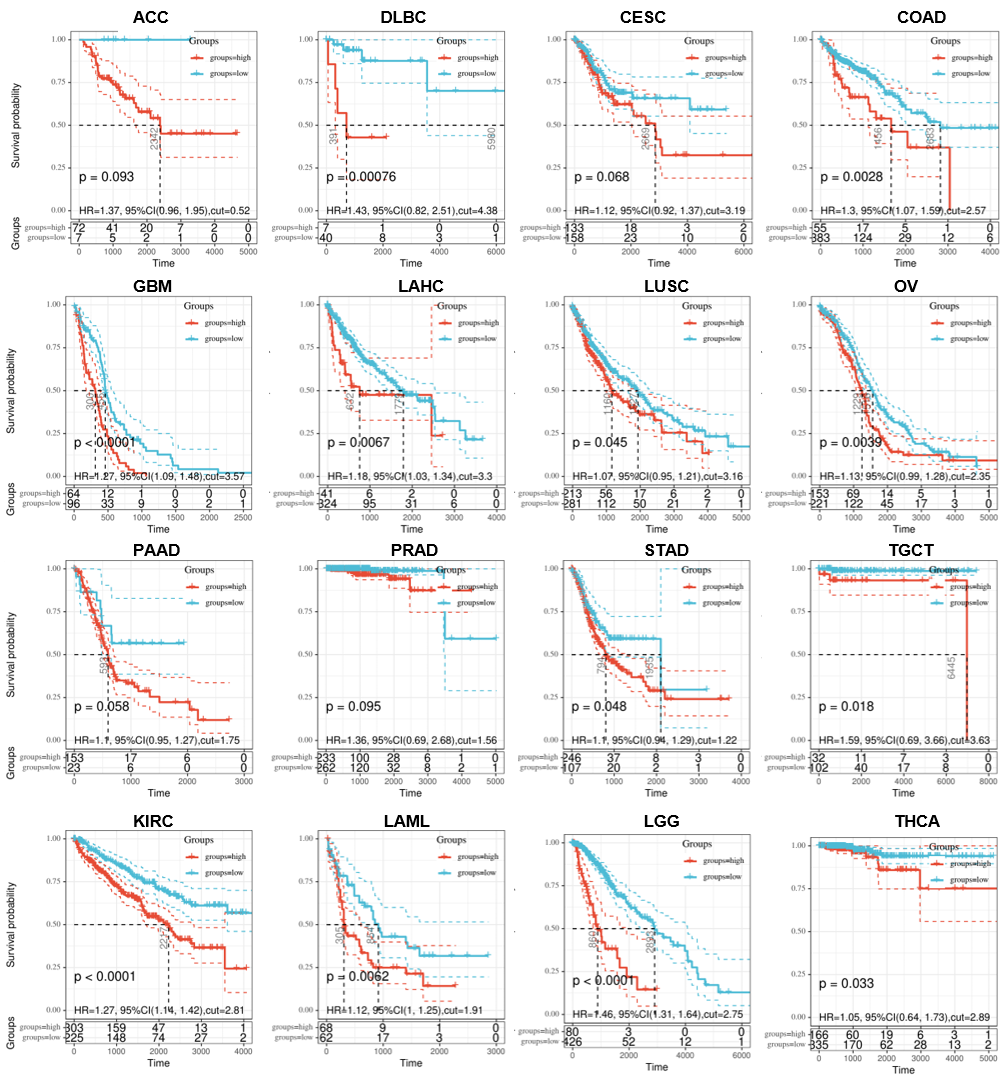


**Figure16 Kaplan–Meier curve analysis indicated that HSPA7 was a risk factor across 16 cancer types.**


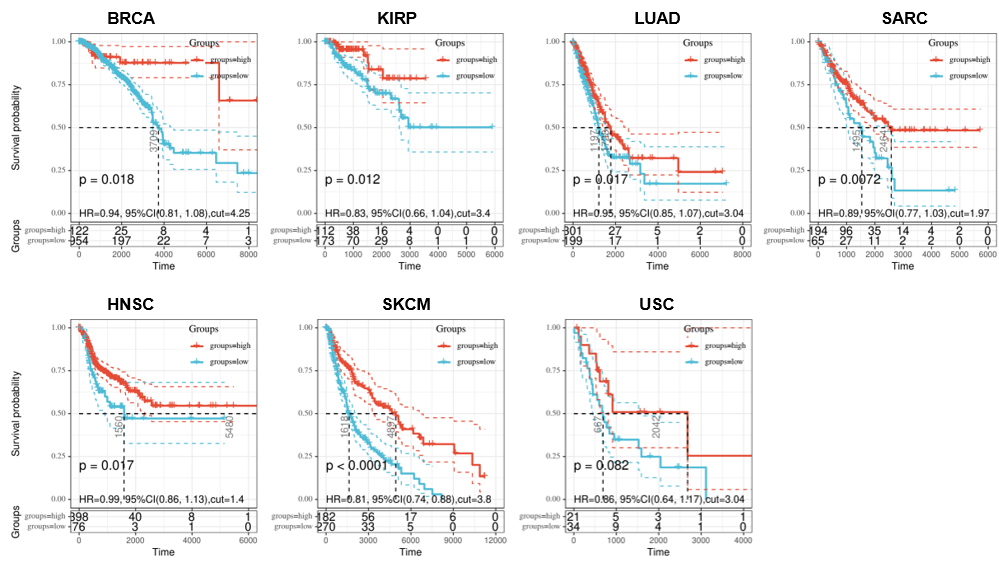


**Figure17 Kaplan–Meier curve analysis indicated that HSPA7 was a favorable factor across 7 cancer types.**


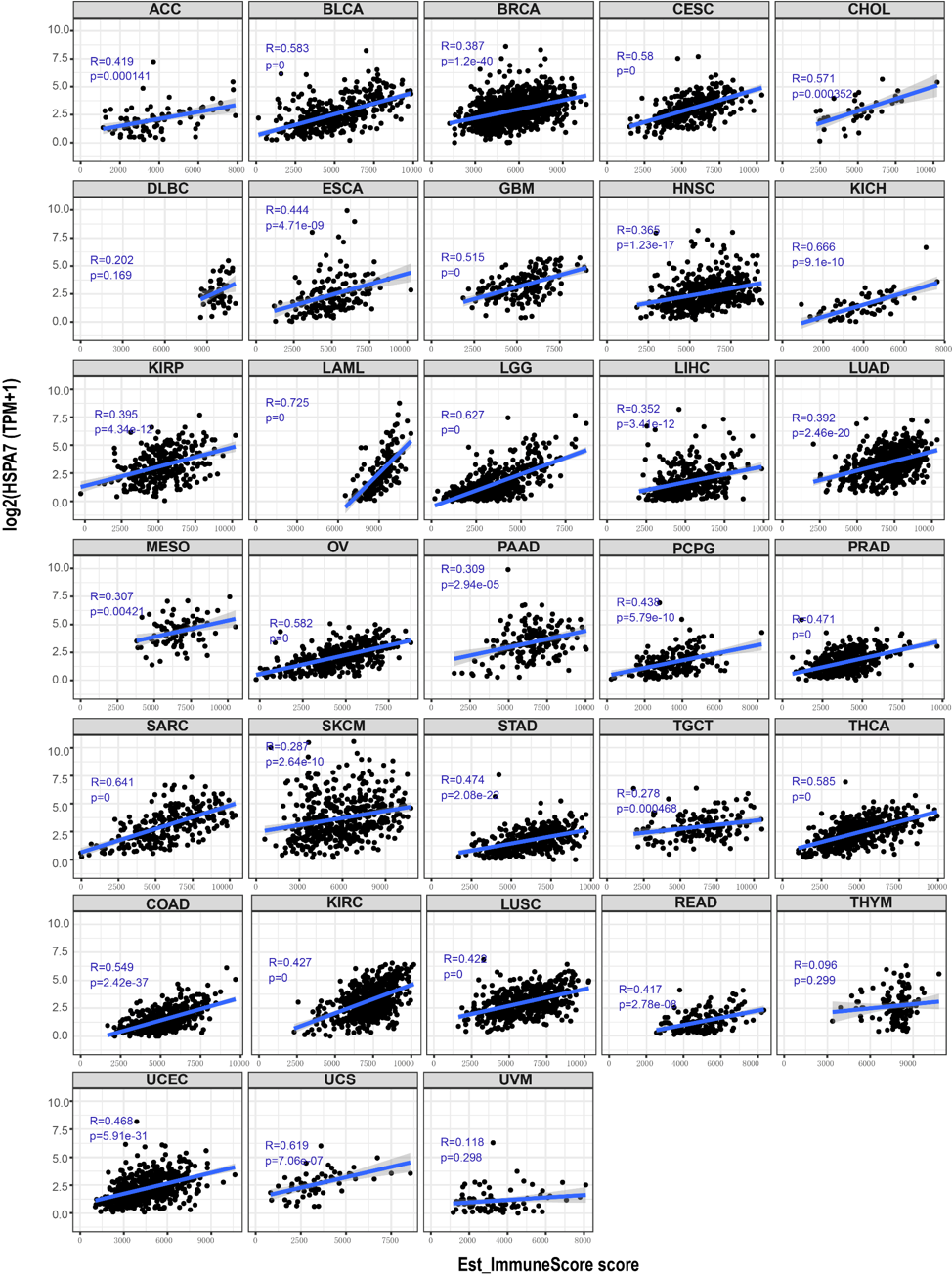


**Figure18 HSPA7 had a significantly positive correlation with the immunescore among all 33 cancer types.**


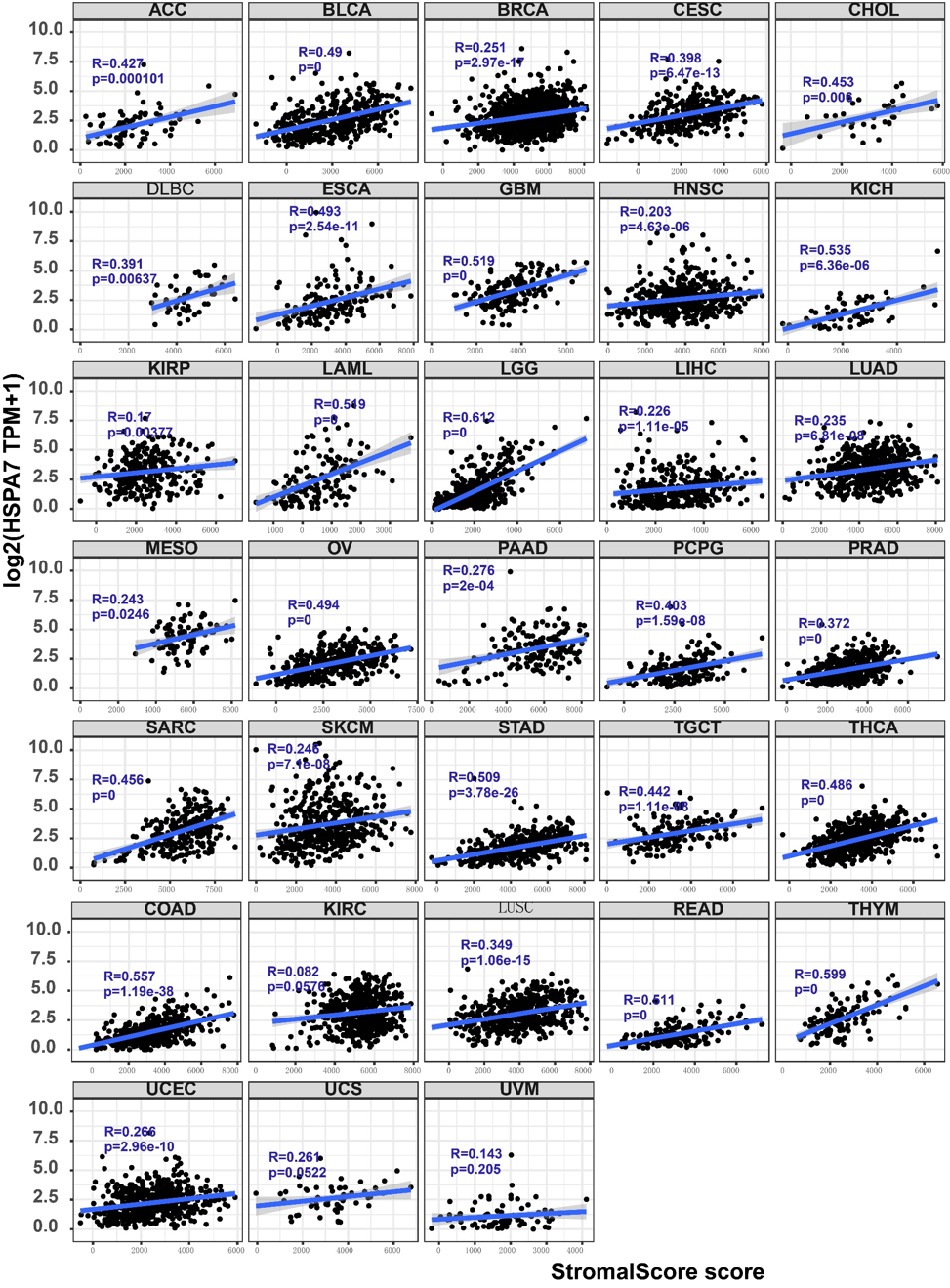


**Figure19 HSPA7 had a significantly positive correlation with the stromalscore among all 33 cancer types.**


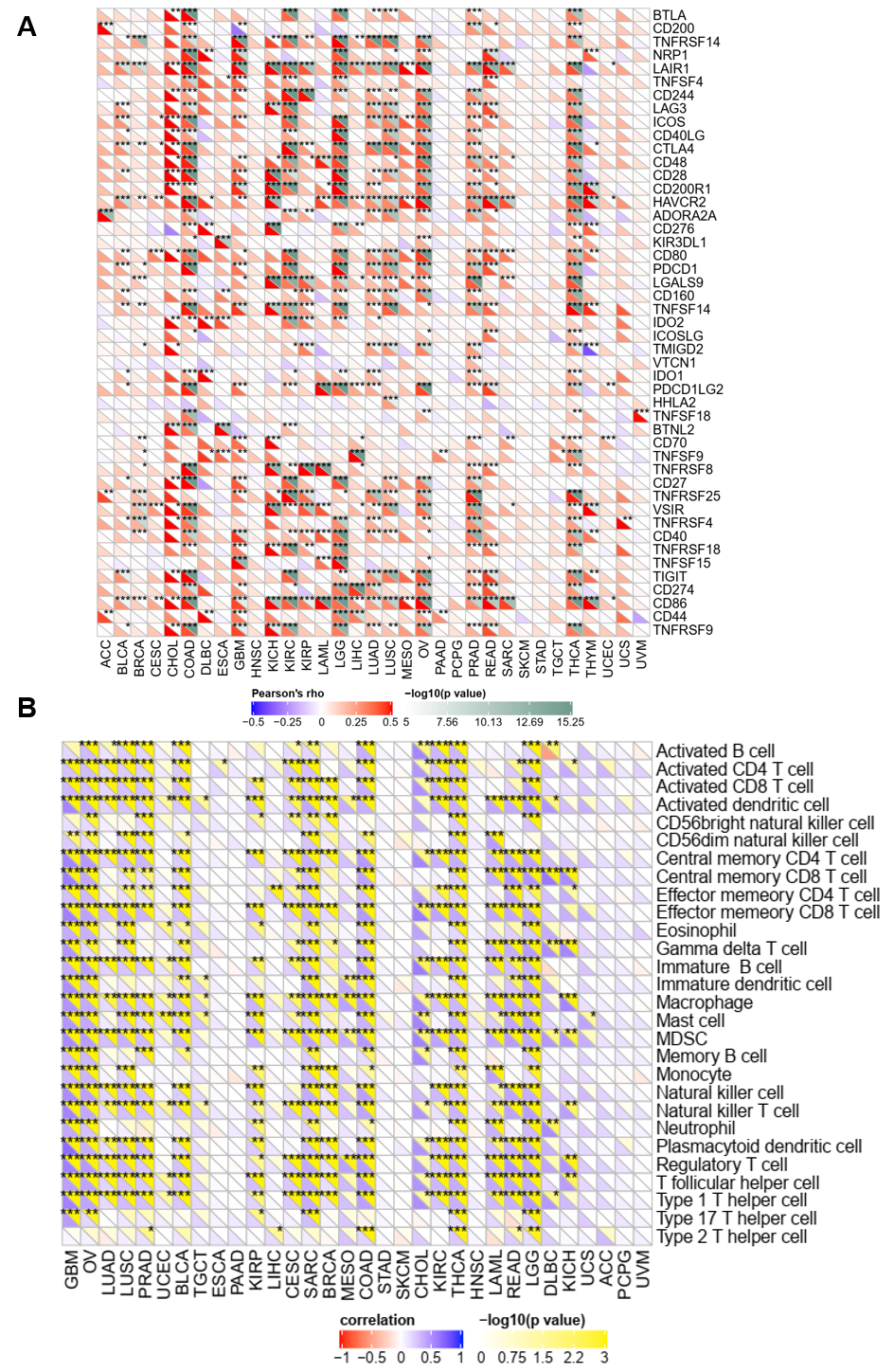


**Figure 20 The correlation of HSAP7 with the immune checkpoints among all 33 cancer types.**

**Supplementary method**

**N^6^-methylated RNA immunoprecipitation-seq (m^6^A-sequencing, m^6^A-seq)**

The m^6^A antibody enriched RNA was sequenced by Novogene (Beijing, China). Briefly, a total of 300 µg RNAs were extracted from the GBM or normal brain tissues. The integrity and concentration of extracted RNAs were detected using an Agilent 2100 bioanalyzer (Agilent) and simpliNano spectrophotometer (GE Healthcare), respectively. Fragmented enriched RNA (~100 nt) was incubated for 2 h at 4℃ with anti-m^6^A polyclonal antibody (Synaptic Systems) in the immunoprecipitation experiment. Then, immunoprecipitated RNAs or input was used for library construction with NEBNext ultra RNA library prepare kit for Illumina (New England Biolabs). The library preparations were sequenced on an Illumina Novaseq or Hiseq platform with a paired-end read length of 150 bp according to vendor’s recommended protocol. The sequencing was carried out with 3 independent biological replicates for each group.

**Quality control**

Raw data (raw reads) of fastq format were firstly processed using fastp (version 0.19.11). In this step, clean data (clean reads) were obtained by removing reads containing adapter, reads containing ploy-N and low-quality reads from raw data. At the same time, Q20, Q30 and GC content the clean data were calculated. All the downstream analyses were based on the clean data with high quality.

**Reads mapping to the reference genome**

Reference genome and gene model annotation files were downloaded from genome website directly. Index of the reference genome was built using BWA v0.7.12 and clean reads were aligned to the reference genome using BWA mem v 0.7.12.

**Peak calling**

The exomePeak（Version 3.8）software was used for peak calling^(1)^. The parameter is as follows: exomepeak(GENE_ANNO_GTF = ｛gtf｝, IP_BAM = ｛ip_bams｝, INPUT_BAM =｛ input_bams｝, OUTPUT_DIR = ｛out_dir｝, EXPERIMENT_NAME = ｛name｝)

The m^6^A peaks were annotated using bedtools（Version 2.25.0, https://bedtools.readthedocs.io/en/latest/）. The parameter is as follows: intersectBed -a ｛peak.bed｝ -b {genome.bed} -wa -wb > {out.bed}.

The deepTools（version 2.4.1）was used for peak distribution analysis^(2)^. The differentially m^6^A peaks were identified by a python script，using fisher test. If at least two samples from each group meet the criteria, it is considered a differential peak. Using the same method, genes associated with different peaks were identified. Sequence motifs enriched in m6A peak regions were identified using Homer（version 4.10）^(3)^. Parameter: findMotifsGenome.pl {peak_file} {genome.fa} {out_dir}

**Identification of differentially expressed genes (DEGs) between immune/stromal distinct groups**

The Stromal Score, Immune Score, ESTIMATE Score, and Tumor Purity were analyzed by ESTIMATE algorithm based on 153 primary GBM RNA-seq data. Then we classified patients into two groups based on the median cutoff expression of immune score and stromal score respectively. DEGs (|FC| ≥1.5，padj ≤ 0.05) between different group were analyzed via “DEseq2” R package.

**Univariate and multivariate Cox regression analysis**

We performed univariate and multivariate Cox regression analysis to calculate the hazard ratios (HR) for HSPA7 and immune cell in TCGA GBM cohort, and the “forestplot” R package was employed to visualize the data of Cox regression analysis of HSPA7.

**Methylated RNA Immunoprecipitation qPCR (MeRIP-qPCR)**.

MeRIP assay was performed by using a Magna RIP RNA-Binding Protein Immunoprecipitation Kit (Millipore), according to the manufacturer’s instructions. Briefly, U251 GBM cells, transfected by NC or shRNA for knockdown METTL3, were harvested and lysed in RIP lysis buffer on ice for 30 min. After centrifugation, the supernatant was incubated with 30 μl of protein-A/G agarose beads (Roche, USA) and m^6^A antibodies (Synaptic Systems, #202003/202011). After overnight incubation, the immune complexes were centrifuged then washed six times with washing buffer. The immunoprecipitated HSPA7 RNA was applied to qRT-PCR analysis. The primer sequences of HSPA7 were shown as below:

HSPA7-F: CCAAGGACAACAACCTGCTG

HSAP7-R: CCCTTGTCATTGGTGATCTTGTT

**References**

1. Meng J, Cui X, Rao M, Chen Y, Huang Y. Exome-based analysis for RNA epigenome sequencing data. *Bioinformatics (Oxford, England)* (2013) 29(12):1565-7. doi: 10.1093/bioinformatics/btt171. PubMed PMID: 23589649.

2. Ramírez F, Ryan D, Grüning B, Bhardwaj V, Kilpert F, Richter A, et al. deepTools2: a next generation web server for deep-sequencing data analysis. *Nucleic acids research* (2016) 44:W160-5. doi: 10.1093/nar/gkw257. PubMed PMID: 27079975.

3. Sven, Heinz, and, Christopher, Benner, and, et al. Simple Combinations of Lineage-Determining Transcription Factors Prime cis-Regulatory Elements Required for Macrophage and B Cell Identities. *Molecular Cell* (2010).
